# Pharmacological mechanism of natural antidepressants: the role of epigenetic modifications

**DOI:** 10.3389/fphar.2025.1616322

**Published:** 2025-08-29

**Authors:** Yitong Lu, Ruirui Shang, Xia Zhong, Jingbo Shi, Guangheng Zhang, Weijie Zhao, Jing Teng

**Affiliations:** ^1^ First Clinical Medical College, Shandong University of Traditional Chinese Medicine, Jinan, China; ^2^ College of Rehabilitation Medicine, Shandong University of Traditional Chinese Medicine, Jinan, China; ^3^ Institute of Child and Adolescent Health, School of Public Health, Peking University, Beijing, China

**Keywords:** epigenetic modifications, depression, natural products, molecular mechanism, antidepressant

## Abstract

MDD is a complex mental illness shaped by the interplay between genetic vulnerability and environmental triggers. Its underlying pathophysiological processes are now understood to be influenced by epigenetic mechanisms. Growing evidence points to critical roles for DNA methylation, histone modification, and ncRNAs in driving transcriptional dysregulation within key brain regions implicated in MDD. These epigenetic alterations may underlie the persistent impairments in neuroplasticity following environmental stress exposure. NPs, renowned for their multi-target properties, have demonstrated promise in modulating epigenetic processes. However, a systematic synthesis of their regulatory roles, mechanisms, and therapeutic potential remains incomplete. This review aims to bridge this gap by integrating evidence from PubMed, Web of Science, and Embase databases to elucidate the role of epigenetic modifications in the pathogenesis and progression of MDD, dissect the mechanisms through which NPs exert antidepressant effects via epigenetic regulation, and highlight current research limitations while proposing strategies for translational applications in both preclinical and clinical settings.

## 1 Introduction

Major depressive disorder (MDD), is a psychiatric disorder characterized by persistent low mood, loss of interest, and cognitive dysfunction. Global epidemiological data show that MDD affects over 350 million people, with a lifetime prevalence rate as high as 15%–20%, and its disability rate ranks second among all diseases (Gore et al., 2011). The World Health Organization predicts that by 2030, MDD will become the leading contributor to the global disease burden, causing over one trillion dollars in economic losses annually (Malhi and Mann, 2018). Current clinical treatments mainly rely on first-line drugs such as selective serotonin reuptake inhibitors (SSRIs), but there are significant limitations: approximately 30%–50% of patients do not respond to existing medications, and issues like delayed onset of action, side effects (sexual dysfunction, weight gain), and withdrawal syndromes are prominent (Gill et al., 2020; Rothmore, 2020). Moreover, traditional monoamine neurotransmitter theories have failed to fully explain the heterogeneity and recurrence mechanisms of MDD. Therefore, breaking through the current treatment framework to develop new antidepressants that feature rapid onset, high safety, and multi-target intervention has become an urgent need in the field of psychopharmacology.

Natural products (NPs), as a diverse pool of secondary metabolites derived from plants, microbes, and marine organisms, exhibit far greater structural complexity than synthetic compound libraries (Penner-Goeke and Binder, 2019). In the field of antidepressant research, flavonoids, terpenoids, and alkaloids have demonstrated a wide range of pharmacological activities, including the regulation of neuroplasticity and suppression of neuroinflammation (Caruso et al., 2022). Critically, NPs often act through multi-target synergistic mechanisms that mimic the body’s intrinsic self-healing processes, offering new insights into the complex pathophysiology of MDD (Zhang et al., 2020). However, the chemical complexity of NPs also poses challenges, such as potential toxicity and poorly defined mechanisms of action, which require systematic elucidation through modern pharmacological approaches.

Epigenetics refers to the regulation of gene expression without alterations in the underlying Deoxyribonucleic Acid (DNA) sequence (Tsou et al., 2021). Major epigenetic mechanisms include DNA methylation, histone modifications, and non-coding RNAs (ncRNAs)-mediated regulation. These modifications play critical roles in controlling gene expression and influence cell differentiation, development, and function. In the context of MDD, the pathogenesis is believed to involve dysregulation of genes related to neurotransmitter synthesis, receptor signaling, transporter activity, and hypothalamic–pituitary–adrenal (HPA) axis function. Recent evidence suggests that epigenetic mechanisms contribute significantly to the development and progression of MDD by modulating the expression and translation of these key genes (Buschdorf and Meaney, 2016; Karpova, 2014). Consequently, epigenetic-based strategies are increasingly recognized as promising targets for therapeutic intervention (Yuan et al., 2023). However, there remains a lack of comprehensive reviews that systematically explore the pathophysiological connections between epigenetic regulation and MDD, as well as the potential of NPs-based interventions to modulate these epigenetic pathways.

In this review, we systematically summarize and analyze the mechanisms by which epigenetic modifications contribute to MDD, and explore the potential and application prospects of NPs in antidepressant treatments through the regulation of these modifications. We hope that this review provides new insights and directions for the development of antidepressant drugs, thereby facilitating the creation of more effective and safer therapies for MDD.

## 2 Review methodology

**FIGURE 1:**
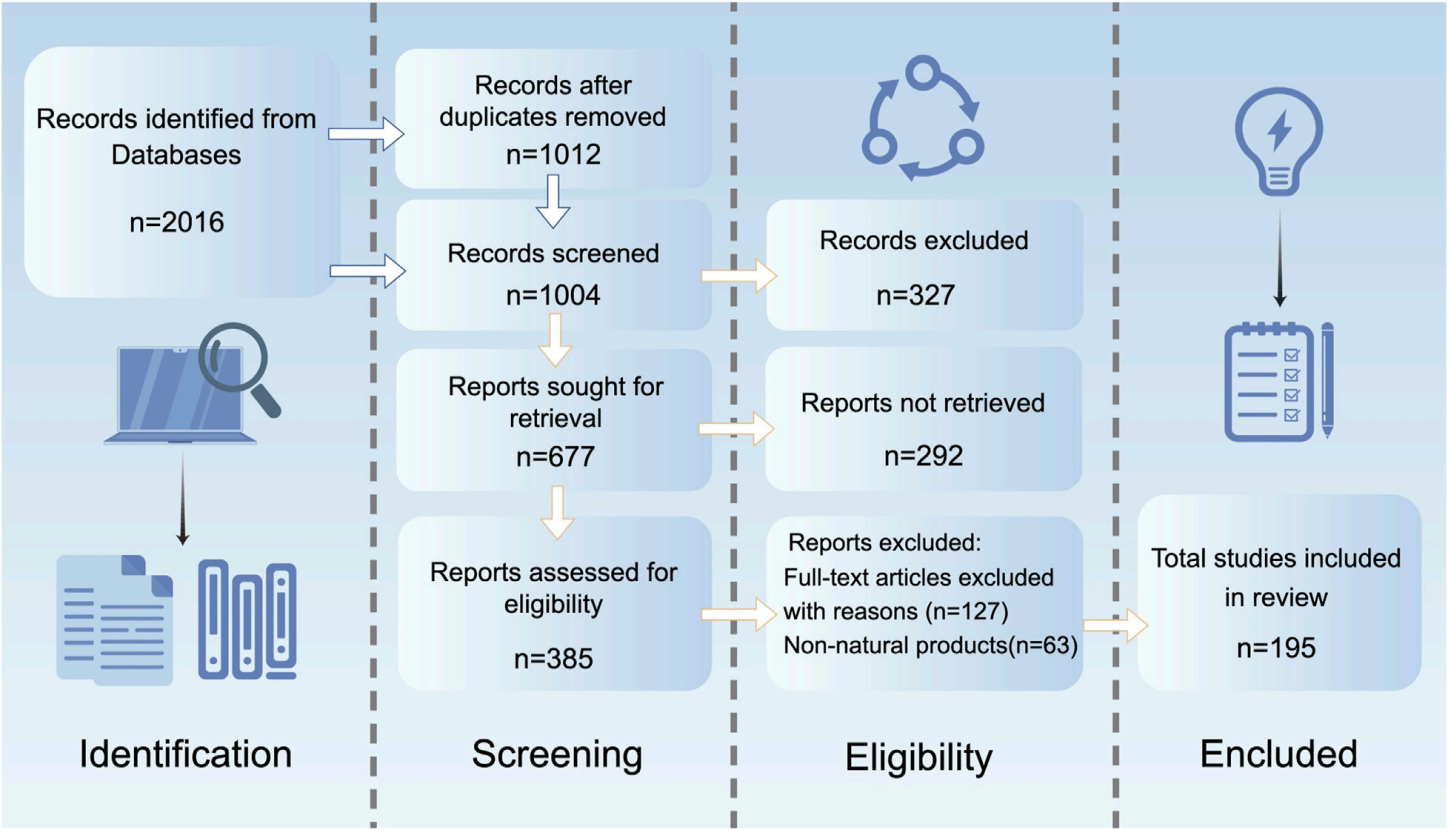
The literature search and screening flowchart.

This study adheres to the Preferred Reporting Items for Systematic Reviews and Meta-Analyses guidelines, conducting a systematic search of studies related to NPs intervening in MDD via epigenetic mechanisms. A systematic search was conducted in PubMed, Web of Science, Embase, and ScienceDirect for studies published from January 2000 to March 2025. The search strategy combined Medical Subject Headings terms and free-text words, including “epigenetic modification,” “DNA methylation,” “histone acetylation,” “non-coding RNA,” “natural products,” “antidepressant,” “depression,” “herb,” “herbal medicine,” “small-molecule drugs,” “flavonoids,” “terpenoids,” and “saponins,” connected through Boolean operators (AND/OR). Inclusion criteria were: ①original research articles published in English; ②studies based on animal or cellular models of MDD; ③investigations clearly exploring the molecular mechanisms by which NPs exert antidepressant effects through epigenetic pathways such as DNA methylation, histone modifications, and non-coding RNA. Exclusion criteria included: ①non-English literature; ②reviews, conference abstracts, case reports, and other grey literature; ③duplicate publications; ④studies not involving NPs or failing to specify epigenetic regulatory mechanisms (e.g., those only describing behavioral outcomes or changes in monoamine neurotransmitters); ⑤interventions involving synthetic drugs or compounds not derived from natural sources. Two researchers independently conducted the literature screening, initially excluding studies that clearly did not meet the criteria based on titles and abstracts. Subsequently, they performed a full-text review of the remaining articles and resolved discrepancies through cross-checking. Ultimately, 189 articles were included for systematic analysis (Figure 1).

## 3 Overview of epigenetic mechanisms

**FIGURE 2:**
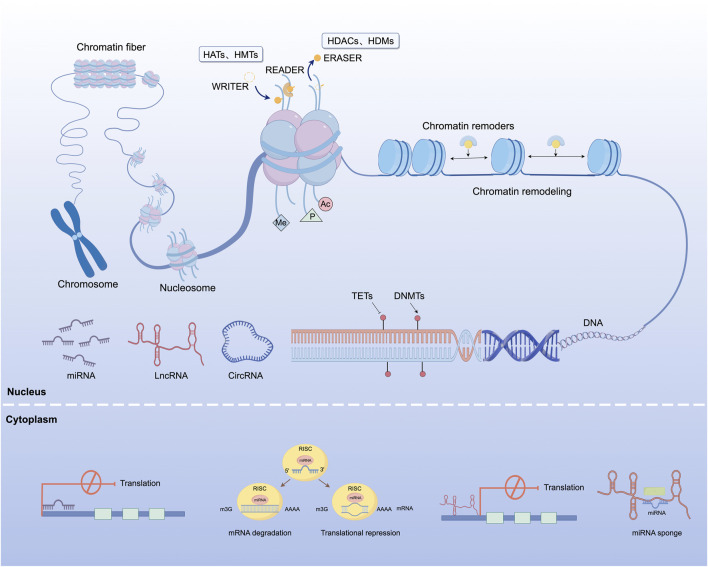
Overview of epigenetic regulatory mechanisms involved in gene expression. This figure illustrates the major epigenetic processes involved in transcriptional regulation. DNA methylation and demethylation are carried out by DNMTs and TETs, respectively. Histone modifications such as acetylation and methylation are catalyzed by HATs and HMTs, removed by HDACs and HDMs, and subsequently recognized by specific chromatin-binding regulatory proteins. Chromatin remodeling is mediated by adenosine triphosphate-dependent remodeling complexes that reposition nucleosomes and modulate chromatin accessibility. In the cytoplasm, ncRNAs including miRNAs, lncRNAs, and circRNAs contribute to post-transcriptional regulation through messenger RNA degradation, translational repression, and miRNA sequestration via competing endogenous RNA activity. These regulatory effects are predominantly mediated by the RISC. HATs, histone acetyltransferases; HMTs, histone methyltransferases; HDACs, histone deacetylases; HDMs, histone demethylases; DNMTs, DNA methyltransferases; TETs, ten-eleven translocation enzymes; miRNA, microRNA; lncRNA, long non-coding RNA; circRNA, circular RNA; RISC, RNA-induced silencing complex.

Epigenetic regulation refers to a group of mechanisms that control gene function without changing the nucleotide sequence of DNA. These mechanisms include, but are not limited to, chemical modifications such as DNA methylation, histone modification, and the actions of ncRNAs. These epigenetic changes can modulate gene transcription through structural changes in chromatin, and are often reversible and inheritable through cell divisions. They play essential roles in development, cellular function, and adaptive responses to environmental signals. As a result, epigenetic mechanisms are critical in maintaining genomic stability and influencing health and disease trajectories (Figure 2).

### 3.1 DNA methylation

DNA methylation refers to the covalent addition of a methyl group to the 5-carbon position of cytosine within Cytosine-phosphate-Guanine (CpG) dinucleotides, catalyzed by DNA methyltransferases (DNMTs). As a core mechanism of epigenetic regulation, this dynamic process is governed by the DNMT family. DNMT3a and DNMT3b are chiefly involved in establishing new DNA methylation marks, while DNMT1 preserves pre-existing methylation patterns during DNA replication. These enzymatic activities function in concert with demethylating regulators, notably the ten-eleven translocation (TET) protein family (Mattei et al., 2022). Within the genome, hypermethylation of CpG islands in promoter regions is typically associated with gene silencing. In contrast, methylation in gene bodies may facilitate transcriptional elongation, and methylation of repetitive elements plays a critical role in maintaining genomic stability (Jones, 2012).

In the brain, DNA methylation is highly dynamic and sensitive to factors such as aging, environmental exposures, and neuronal activity (Lister et al., 2013). During early embryonic development, DNA methylation plays a crucial role in the lineage commitment of neural progenitor cells. *De novo* methyltransferases such as DNMT3a and DNMT3b are essential for silencing pluripotency genes and activating lineage-specific programs. Loss of DNMT function impairs neural differentiation and disrupts epigenetic patterning (Smith and Meissner, 2013). These findings highlight DNA methylation as a key regulator of neurodevelopmental fate decisions. In the adult brain, particularly in the hippocampus, localized DNA methylation reprogramming contributes to learning and memory formation. Environmental stimuli or cognitive tasks can induce methylation changes at specific loci, regulating gene expression associated with synaptic plasticity and memory consolidation (Zocher et al., 2021). Environmental stressors such as chronic stress or nutrient deprivation can activate glucocorticoid receptor (GR) signaling pathways, which in turn promote the recruitment of DNMTs to stress-responsive gene promoters. For example, early-life adversity has been shown to increase methylation at the promoter of the GR gene nuclear receptor subfamily 3 group C member 1 (NR3C1) in the hippocampus, thereby suppressing its expression and disrupting negative feedback regulation of the HPA axis (Bakusic et al., 2021).

### 3.2 Histone modification

Histones, the core components of chromatin, are responsible for packaging genomic DNA into compact chromatin structures. Histone modifications, which involve covalent chemical changes at specific amino acid residues such as acetylation, methylation, phosphorylation, and ubiquitination, play dynamic roles in regulating chromatin organization. These modifications influence chromatin condensation and DNA accessibility, thereby modulating gene expression patterns. They function as essential epigenetic mechanisms that control a wide range of physiological and developmental processes in eukaryotic cells. Among these modifications, histone acetylation and methylation have been the most extensively studied and are recognized as major regulators of transcriptional activity.

Among all histone modifications, acetylation is a highly dynamic mark that is closely associated with transcriptional activation. This process is catalyzed by lysine acetyltransferases (KATs), which transfer the acetyl group from acetyl-CoA to the ε-amino group of lysine residues on histones. This modification neutralizes the positive charge of histones, thereby weakening the electrostatic interactions between histones and DNA. As a result, chromatin structure becomes more relaxed, enhancing DNA accessibility and facilitating transcriptional initiation (Shvedunova and Akhtar, 2022). Histone acetylation predominantly occurs on lysine residues at the N-terminal tails of histones H3 and H4. Common modification sites include histone H3 lysine 9 acetylation (H3K9ac), histone H3 lysine 14 acetylation (H3K14ac). These marks are strongly associated with transcriptionally active chromatin and are widely used as epigenetic indicators in functional studies (Kouzarides, 2007). In contrast to KAT-mediated acetylation, histone deacetylases (HDACs) remove acetyl groups from lysine residues, thereby strengthening histone-DNA interactions, promoting chromatin condensation, and repressing gene expression (D’Mello, 2020).

Histone methylation typically occurs on lysine and arginine residues and is catalyzed by histone methyltransferases (HMTs). The functional outcome of this modification depends on both the specific residue involved and the number of methyl groups added. Methylation can occur in the form of mono, di, or trimethylation, each with distinct regulatory effects on gene expression (Yang et al., 2020). For instance, trimethylation at histone H3 lysine 4 trimethylation (H3K4me3) is associated with active gene promoters and facilitates transcription. In contrast, methylation at histone H3 lysine 27 methylation (H3K27me) and H3K27me3 is linked to gene repression and is typically enriched in heterochromatic regions and transcriptionally silent loci (Di Nisio et al., 2021).

### 3.3 NcRNAs

NcRNAs constitute a class of functionally diverse ribonucleic acid (RNA) molecules that do not encode proteins but play critical regulatory roles, accounting for over 60% of the mammalian transcriptome. NcRNAs can be broadly classified according to their sequence length and biological function into three major types: long non-coding RNAs (lncRNAs, typically exceeding 200 nucleotides), microRNAs (miRNAs, approximately 18–25 nucleotides), and circular RNAs (circRNAs) (Reik, 2007; Zaratiegui et al., 2007). These molecules dynamically regulate chromatin architecture, genomic stability, and post-transcriptional modifications through interactions with DNA, RNA, proteins, or chromatin complexes, thereby influencing cellular differentiation, development, and disease progression via epigenetic mechanisms. LncRNAs can recruit polycomb repressive complex 2 to deposit H3K27me3 marks at target gene promoters, leading to transcriptional silencing (Guo et al., 2021). MiRNAs bind to complementary seed sequences within the 3′untranslated regions (3′UTRs) of target mRNAs, mediating translational repression or degradation (Bartel, 2009). This enables miRNAs to fine-tune gene expression networks, as exemplified by region-specific miRNA ensembles in neurons that regulate local protein synthesis to modulate synaptic homeostasis and plasticity (Martins and Schratt, 2021). Owing to their covalently closed circular structure, circRNAs exhibit enhanced stability and function as miRNA sponges. By sequestering miRNAs (e.g., circRNA CDR1as binding miR-7), they relieve miRNA-mediated suppression of target mRNAs (Misir et al., 2022; Yang et al., 2016). Additionally, circRNAs interact with RNA-binding proteins to form novel regulatory circuits. For example, CDR1 as modulates synaptic plasticity through both miR-7 sponging and direct protein interactions (Mehta et al., 2022).

### 3.4 Chromatin remodeling

Unlike DNA methylation and histone modifications that regulate gene expression through chemical alterations of chromatin, chromatin remodeling primarily operates via physical restructuring of chromatin architecture. The core mechanism involves Adenosine Triphosphate (ATP)-dependent chromatin remodelers that utilize energy from ATP binding/hydrolysis to modulate nucleosome positioning through sliding, eviction, or histone variant replacement, thereby regulating transcriptional accessibility. Depending on the specificity of different ATPase subunits, ATP-dependent chromatin remodeling complexes are primarily classified into four categories: the switching defective/sucrose nonfermenting (SWI/SNF) family, interphase structure whirlpool (ISWI) family, inositol requiring protein 80 (INO80) family, and chromodomain helicase DNA-binding (CHD) family (Flaus et al., 2006). Chromatin remodeling complexes exhibit distinct regulatory mechanisms based on their subunit composition. SWI/SNF-family proteins mediate chromatin accessibility by displacing nucleosomes to create nucleosome-free regions, facilitating transcriptional activation (Whitehouse et al., 1999). ISWI-family complexes regulate nucleosomal spacing to maintain chromatin structural integrity (Ito et al., 1997), while CHD-family members coordinate transcriptional processes through nucleosome binding and interactions with transcription elongation and chromatin modification factors (Reyes et al., 2021); INO80-family remodelers specialize in histone variant exchange to dynamically modulate chromatin states (Papamichos-Chronakis et al., 2011). Additionally, chromatin remodeling activity is regulated by multiple mechanisms, including auto-inhibition, histone modifications, and auxiliary subunit functions (Wang et al., 2021).

## 4 Pathologic connection between epigenetic mechanisms and MDD

**FIGURE 3:**
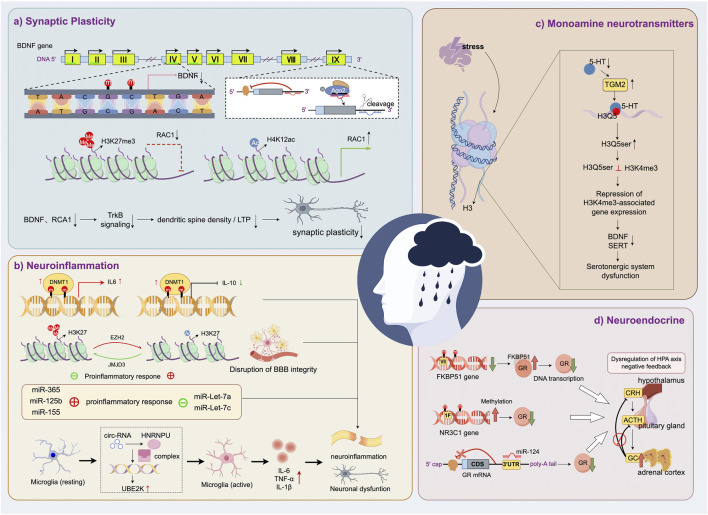
Epigenetic regulation of MDD-related pathophysiological mechanisms. **(a)** Synaptic plasticity is impaired by epigenetic downregulation of BDNF and RAC1. **(b)** Neuroinflammation is promoted through epigenetic control of cytokine expression and microglial activation. **(b)** Serotonergic dysfunction results from stress-induced disruption of histone serotonylation and gene repression. **(d)** HPA axis imbalance is driven by epigenetic regulation of glucocorticoid receptor signaling.

MDD arises from the complex interplay of biological, psychological, and social determinants, with epigenetics emerging as a pivotal regulatory layer elucidating its underlying mechanisms. Key epigenetic modifications, including DNA methylation, histone post-translational modifications, chromatin remodeling, ncRNAs, and stress-responsive pathways, orchestrate fine-tuned regulation of gene expression without altering DNA sequences, thereby modulating brain structure and function. Accumulating evidence demonstrates that these mechanisms critically regulate neurodevelopment, synaptic plasticity, and adaptive responses to environmental stressors. In subsequent sections, we systematically dissect how specific epigenetic alterations contribute to MDD pathophysiology, with emphasis on their dynamic interplay and disease-specific roles (Figure 3).

### 4.1 DNA methylation in MDD

#### 4.1.1 Childhood adversity as a key factor in methylation-driven MDD risk

DNA methylation is increasingly recognized as a critical molecular bridge connecting environmental risk exposures with genetic vulnerability in MDD. Large-scale cohort studies demonstrate that childhood adversities (e.g., maltreatment, neglect, household dysfunction) confer elevated MDD risk through enduring methylation imprints. A landmark epigenome-wide association study investigating blood-derived DNA methylation patterns linking seven childhood adversity types (age 0–7) with adolescent depressive symptoms (mean age 10.6) revealed adversity-associated methylation alterations at 70 CpG sites. These loci collectively mediated 10%–73% of adversity-MDD symptom associations (Lussier et al., 2024). Hypermethylation at 39 CpG sites exhibited protective effects, suggesting DNA methylation may partially buffer psychological trauma through compensatory mechanisms, a finding highlighting novel directions for understanding MDD resilience.

#### 4.1.2 Changes in DNMTs and TETs in MDD

The dynamic process of DNA methylation requires the involvement of DNMTs and TETs, with the expression levels and activities of these enzymes playing a crucial role in the DNA methylation process. Their activities also regulate mood behaviors and are closely related to the onset and progression of MDD. Animal experiments have found that chronic social defeat stress (CSDS) increases the levels of DNMT3a in the nucleus accumbens (NAc) of mice, and overexpression of DNMT3a promotes depressive-like behaviors, while local administration of the DNMT inhibitor reverses such behaviors (LaPlant et al., 2010). A similar result was observed in a clinical study (Hodes et al., 2015). Previous studies have demonstrated that DNMTs also undergo stress-related expression changes in other key brain regions. In the prefrontal cortex (PFC) and hippocampus, DNMT1/3a are upregulated in the chronic unpredictable mild stress (CUMS) model, and their expression can be reversed by antidepressant treatment (Cheng et al., 2023). In contrast, the amygdala shows a distinct expression pattern. *Postmortem* analyses have revealed reduced DNMT1/3B mRNA levels in the amygdala of patients with MDD (Poulter et al., 2008). However, in animal models, DNMT1 expression is increased in the central nucleus of the amygdala following CSDS, with this change observed exclusively in female mice, suggesting a sex-specific regulatory mechanism in this region (Wright et al., 2017).

Furthermore, studies have shown that TET1 knockout mice exhibit resistance to chronic restraint stress, whereas TET2 knockout mice show hypersensitivity to stress (Cheng et al., 2018). When TET1 protein is overexpressed in the hippocampus of mice, it upregulates the expression levels of Delta-like canonical Notch ligand 3 and Notch1 proteins, promoting hippocampal neurogenesis and alleviating depressive-like behaviors (Shuang et al., 2024). Under stress conditions, abnormal expression and decreased nuclear translocation of TET2 lead to a reduction in 5-hydroxymethylcytosine levels and dysregulated gene expression, increasing susceptibility to MDD in mice (Zhang et al., 2021a). Additionally, the rapid antidepressant effect of ascorbic acid is mediated by the activation of DNA demethylation catalyzed by TET1 and TET2 (Han et al., 2022). Similarly, TET enzymes also exhibit region-specific roles in depression-like behavior. CSDS leads to a downregulation of TET1 expression exclusively in the NAc of susceptible mice, while selective deletion of TET1 in the NAc induces antidepressant-like effects. Mechanistically, TET1 deficiency relieves transcriptional repression of immune-related gene clusters via demethylation, resulting in the upregulation of genes that closely overlap with the expression profile of resilient mice. These findings suggest that TET1 in the NAc may function as a negative regulator of stress responses (Feng et al., 2017).

#### 4.1.3 The methylation dysregulation of key genes associated with MDD

Brain-derived neurotrophic factor (BDNF), a widely expressed neurotrophin critical for synaptic transmission and plasticity, plays pivotal roles in late-stage neurodevelopment and psychiatric disorder pathogenesis (Zelada et al., 2023). Clinical studies consistently report reduced BDNF expression and protein levels in peripheral blood and postmortem brain tissues of MDD patients, positioning BDNF deficiency as a key etiological factor (Gelle et al., 2021). The synthesis of BDNF in neurons, which is significantly diminished in MDD, is closely associated with elevated methylation levels in the BDNF promoter region, and given that the methylation status of the BDNF gene has been implicated as a crucial factor in the pathogenesis of MDD, it is increasingly recognized as a potential biomarker for the disorder. Li et al. identified elevated methylation levels at two CpG dinucleotides (BDNF133 and BDNF134) within exon VI of the BDNF gene in MDD patients relative to healthy controls (Li et al., 2021a). In contrast, a recent study demonstrated reduced pre-treatment methylation in the promoter region of exon IV in adolescents with MDD compared to non-affected individuals (Zwolińska et al., 2024). These discordant findings highlight the context-dependent nature of BDNF methylation patterns, suggesting substantial variability influenced by exon specificity, developmental stage, sex, environmental exposures. Consequently, the clinical utility of BDNF methylation as a MDD biomarker necessitates multi-faceted validation through large-scale, population-diverse cohort studies incorporating multi-omics approaches to account for these confounding variables. On the other hand, BDNF promoter methylation appears to be associated with brain structural alterations in MDD patients. Choi et al. found a significant negative correlation between the methylation status of the BDNF promoter region and the integrity of the right anterior corona radiata white matter, which is involved in emotional and cognitive control networks implicated in the pathophysiology of MDD. This suggests that BDNF gene methylation may contribute to the pathogenesis of MDD by regulating white matter structural integrity (Choi et al., 2015). Meanwhile, reduced cortical thickness in the prefrontal and occipital regions of MDD patients was associated with increased methylation levels at the BDNF promoter in these areas (Na et al., 2016).

NR3C1, a critical regulator of stress responses, modulates GR levels and HPA axis activity. Hyperactivation of the HPA axis and elevated glucocorticoid (GC) levels represent hallmark pathophysiological features of MDD. Experimental evidence reveals that two GC dinucleotide pairs within the human NR3C1 coding sequence are typically unmethylated under normal conditions. However, stress-induced methylation at these sites suppresses NR3C1 transcription, reduces GR mRNA levels, elevates cortisol concentrations, and impairs HPA axis negative feedback, ultimately driving HPA hyperactivation and depressive pathogenesis (Bustamante et al., 2016). In rodent models, the maternal tactile stimulation (licking/grooming) during postnatal day 1 reduces HPA reactivity and alleviates anxiety-like behaviors in offspring. This protective effect is mediated by demethylation of the NR3C1 exon 1–7 promoter in the hippocampus (Murgatroyd et al., 2015). Moreover, Early-life stress (e.g., childhood trauma, neglect) is a well-established risk factor for MDD (Nelson et al., 2017). Studies demonstrating that NR3C1 undergoes stress-induced epigenetic modifications, particularly DNA methylation, which confers lifelong MDD susceptibility (Keller et al., 2017). Adults with MDD and childhood maltreatment (CM) histories exhibit elevated NR3C1 promoter methylation, with methylation levels positively correlating with CM severity and subtype (Perroud et al., 2011).

FKBP5 encodes FK506-binding protein 51 (FKBP51), a critical negative regulator of the GR signaling pathway. By interfering with GR nuclear translocation and function, FKBP51 modulates the activity of the HPA axis, thereby affecting the balance of the stress response. Dysregulation of the HPA axis is a core pathophysiological mechanism in MDD, and polymorphisms in the FKBP5 gene have been shown to confer increased vulnerability to MDD, particularly among individuals exposed to early-life trauma or chronic stress (Klengel et al., 2012). Epigenetically, methylation at specific CpG sites within intron 7 of FKBP5 (e.g., cg25563198 in the TSS1500 region) has been identified as a regulatory element of transcriptional activity. Hypomethylation in this region is associated with elevated FKBP5 expression, which enhances its inhibition of GR activity, leading to impaired negative feedback of the HPA axis, GC resistance, and prolonged stress responses (Tang et al., 2024). This epigenetic alteration may interact with specific risk genotypes such as rs1360780, further modulating gene-environment interactions in the development of depressive pathology. Collectively, the hypomethylation of FKBP5 intron 7 represents a crucial mechanism linking environmental stress exposure to HPA axis dysregulation and increased risk for MDD (Klinger-König et al., 2019).

The Solute Carrier Family 6 Member 4 (SLC6A4) gene encodes the serotonin (5-HT) transporter, which plays a key role in regulating 5-HT reuptake and neurotransmission. DNA methylation of the SLC6A4 promoter region has been consistently associated with reduced gene expression and 5-HT dysregulation, which are both implicated in the neurobiology of MDD. Kang et al. reported that individuals with more severe depressive symptoms and a history of childhood adversity showed significantly higher SLC6A4 methylation levels (Kang et al., 2013). Similarly, Bakusic et al. found that hypermethylation of both SLC6A4 and NR3C1 was associated with blunted cortisol reactivity following acute psychosocial stress in MDD patients (Bakusic et al., 2020). This functional attenuation of the HPA axis was especially evident during the recovery phase after social stress exposure. These findings suggest that hypermethylation of stress-related genes may contribute to the pathophysiology of MDD by impairing the dynamic regulation of the stress response. Furthermore, methylation of a specific CpG site in NR3C1 (CpG20) was predictive of poor symptom improvement over an 8-week follow-up, highlighting its potential as a biomarker for treatment response.

### 4.2 Histone modifications in MDD

#### 4.2.1 Histone acetylation

Histone acetylation plays a critical role in regulating chromatin structure and gene transcription. In recent years, it has been widely investigated as a key epigenetic mechanism in the pathogenesis of MDD. In animal models, chronic stress significantly reduces histone acetylation levels in emotion-regulating brain regions such as the hippocampus. This loss of acetylation is often accompanied by downregulation of BDNF expression, impaired synaptic plasticity, and diminished stress resilience, ultimately leading to depression-like behaviors such as social withdrawal and anhedonia (Tsankova et al., 2007). Interestingly, some acetylation marks exhibit a gradual increase during the recovery phase, typically between 15 and 21 days following stress cessation (Montagud-Romero et al., 2016). This observation suggests that the dynamic restoration of histone acetylation may play a central role in the natural remission of depressive symptoms. In addition, metabolic agents such as metformin can enhance histone acetylation via the AMP-activated protein kinase/cAMP response element-binding protein (AMPK/CREB) signaling pathway, leading to increased BDNF expression and behavioral improvement (Fang et al., 2020). Moreover, some studies have shown that in the CSDS model, HDAC7 expression is downregulated in the NAc, leading to reduced histone deacetylation and a relative increase in histone acetylation levels (Qian et al., 2021). Another study reported that CSDS induces a delayed increase in H3K14ac in the mPFC of susceptible mice, with significant elevation observed at 24 h and 10 days after stress, returning to baseline by day 20 (Covington et al., 2015). These findings suggest that histone acetylation is regulated in a region-specific manner across different brain areas in response to stress.

Microglia, as the principal effector cells of neuroinflammation in the central nervous system (CNS), play a crucial role in the pathophysiology of MDD. Elevated expression of HDAC1 in microglia has been shown to suppress the transcription of anti-inflammatory genes such as IL-10 by reducing H3K9ac. At the same time, HDAC1 activity promotes the expression of pro-inflammatory cytokines, including interleukin-6 (IL-6) and tumor necrosis factor-alpha (TNF-α), as well as the activation of the NOD-like receptor family pyrin domain containing 3 (NLRP3) inflammasome, thereby amplifying the neuroinflammatory cascade (Patnala et al., 2017). Elevated HDAC1 activity also promotes the polarization of microglia toward a pro-inflammatory phenotype, thereby strengthening the link between the inflammatory microenvironment and depression-like behaviors. Mechanistic studies have shown that the antidepressant fluoxetine alleviates depressive susceptibility in mice by suppressing HDAC1 expression, activating the phosphoinositide 3-kinase/protein kinase B/mammalian target of rapamycin (PI3K/Akt/mTOR) signaling pathway, and reducing both eukaryotic elongation factor 2 activity and NLRP3 inflammasome expression. Notably, administration of enidone, a selective HDAC1 agonist, completely abolished the anti-inflammatory and antidepressant effects of fluoxetine, confirming the pivotal role of HDAC1 as an epigenetic therapeutic target (Li et al., 2021b).

#### 4.2.2 Histone methylation

The function of histone methylation as a reversible epigenetic mark is highly dependent on the modification site. Among the many histone modifications, H3K4me3 is one of the most widely studied modifications. Early evidence from Uchida et al. demonstrated that H3K4me3 levels at the glial cell line-derived neurotrophic factor promoter were reduced in the ventral striatum of stress-susceptible mice, contributing to altered neurotrophic support in MDD (Uchida et al., 2011). Subsequent findings by Cruceanu et al. revealed excessive enrichment of H3K4me3 at the synapsin 1 (SYN1) promoter in MDD patients, leading to the overexpression of SYN1a and SYN1b and potentially disrupting synaptic plasticity (Cruceanu et al., 2013). More recently, Tseng et al. reported decreased H3K4me3 levels at promoters of toll-like receptor 4 (TLR4) pathway genes in MDD brains, correlating with MDD severity and implicating immune dysregulation as a potential mechanism (Tseng et al., 2023).

In contrast to H3K4me3, which activates gene transcription, other forms of histone methylation, such as histone H3 lysine 9 dimethylation (H3K9me2) and H3K27me3, inhibit gene transcription (Li et al., 2021c; Wang et al., 2023). CSDS-induced MDD models demonstrated aberrantly increased H3K9me2/me3 levels in the NAc, resulting in transcriptional repression of target genes, which impairs neuroplasticity and reward circuitry function, linking to core depressive symptoms such as anhedonia (Pathak et al., 2017). Concurrently, Ras-related C3 botulinum toxin substrate 1 (RAC1), a small guanosine triphosphatase (GTPase) critical for cytoskeletal dynamics, exhibits reduced expression in the NAc, resulting in synaptic structural and functional alterations that are closely linked to depressive-like behaviors. Studies have shown that reduced expression of RAC1 in the NAc of mice exposed to CSDS is associated with increased levels of H3K27me3 (Golden et al., 2013). In stress-induced murine MDD models, Claudin-5 dysregulation was linked to elevated H3K27me3 at its promoter. Enhancer of zeste homolog 2 (EZH2) catalyzed H3K27me3 deposition to repress Claudin-5, thereby compromising blood-brain barrier (BBB) integrity, triggering neuroinflammation, and exacerbating depressive behaviors. EZH2 knockdown or antidepressant treatment reduced H3K27me3 levels, restored Claudin-5 expression, and rescued depressive-like phenotypes (Sun et al., 2024). More recently, polycomb group ring finger 1 (PCGF1), a component of the noncanonical polycomb repressive complex 1, was found to alleviate adolescent MDD by suppressing matrix metallopeptidase 10 (MMP10) transcription in hippocampal microglia. PCGF1 increased the enrichment of both H2AK119ub and H3K27me3 at the MMP10 promoter, thereby repressing nuclear factor kappa-light-chain-enhancer of activated B cells/mitogen-activated protein kinase (NF-κB/MAPK)-mediated inflammatory signaling and improving behavioral outcomes (Li et al., 2025). Collectively, these findings underscore that histone methylation, depending on site-specific patterns and the activity of HMTs, regulates key biological pathways in MDD, including synaptic plasticity, neuroimmune signaling, and BBB integrity.

#### 4.2.3 Histone crotonylation

Histone crotonylation is a novel acyl modification mediated by crotonyl-CoA donors, strongly associated with transcriptional activation. Crotonyl-CoA hydratase (CDYL) suppresses this modification by hydrolyzing crotonyl-CoA. Pioneering work by Huang’s team revealed that CUMS significantly reduces H3K9 crotonylation levels in the prelimbic cortex of mice, concomitant with decreased expression of neuropeptide and synaptic loss. CDYL overexpression in the PL heightened susceptibility to depressive phenotypes, whereas CDYL suppression or crotonate supplementation rescued depressive-like behaviors (Liu et al., 2019a).

#### 4.2.4 Histone monoaminylation

Histone monoaminylation is a recently identified form of epigenetic modification involving the covalent attachment of neurotransmitters such as 5-HT, dopamine, and histamine to the glutamine residue at position 5 of histone H3. This process is typically catalyzed by transglutaminase 2 and results in the formation of the composite mark H3K4me3Q5ser in the presence of H3K4 trimethylation. This dual modification enhances the recruitment of the transcription factor transcription factor IID (TFIID) to chromatin and concurrently suppresses demethylase activity, thereby sustaining the transcriptional activation of key genes (Farrelly et al., 2019).

Recent studies have shown that histone monoaminylation not only represents a downstream extension of neurotransmitter signaling but also directly regulates gene expression. In a CSDS model, stress-susceptible animals displayed dynamic changes in histone H3 lysine 4 trimethylation and glutaminyl-serotonylation at glutamine 5 (H3K4me3Q5ser) levels within the dorsal raphe nucleus, characterized by a marked decrease during the acute phase and abnormal accumulation during the prolonged stress period. These alterations were closely associated with stress-induced behavioral phenotypes, suggesting that this epigenetic mark may contribute to the development of depression-like behaviors. Pharmacological intervention further demonstrated that chronic fluoxetine treatment significantly restored H3K4me3Q5ser levels, which was accompanied by an improvement in depressive-like behaviors. Moreover, virally mediated expression of the H3.3Q5A mutant, which blocks monoaminylation at the Q5 site, also reversed both transcriptional abnormalities and behavioral impairments induced by stress. These findings indicate that H3K4me3Q5ser functions not only as a state-dependent marker but also as an epigenetic regulator of mood-related plasticity. Consistent results have been observed in postmortem brain tissue from individuals with MDD. Patients who had not received antidepressant treatment exhibited significantly lower levels of H3K4me3Q5ser, whereas treated patients showed no significant difference compared to healthy controls. This suggests that H3K4me3Q5ser may serve as a potential biomarker for antidepressant responsiveness (Al-Kachak et al., 2024). Collectively, these findings support a critical epigenetic role for histone monoaminylation, particularly H3K4me3Q5ser, in modulating transcriptional plasticity under stress, which may partly explain the delayed therapeutic effects of conventional antidepressants.

### 4.3 NcRNAs in the epigenetic regulation of MDD

#### 4.3.1 MiRNAs

Among ncRNAs, miRNAs constitute a pivotal epigenetic regulatory system and have emerged as master regulators of neuroplasticity and higher-order brain functions. Multiple studies have identified significant differences in miRNA expression profiles between individuals with MDD and healthy controls. These miRNA alterations are closely associated with the pathophysiological mechanisms of MDD, participating in its pathogenesis through multidimensional molecular pathways.

MiRNAs regulate key proteins involved in monoaminergic neurotransmitter metabolism, thereby modulating synaptic neurotransmitter homeostasis. Notably, miR-16-mediated targeted inhibition of the serotonin transporter (SERT) has been implicated in the pathological dysregulation of the monoaminergic system. CSDS downregulates miR-16 expression in the cerebrospinal fluid and raphe nuclei, leading to excessive SERT protein accumulation and enhanced synaptic 5-HT reuptake, which ultimately elicits depression-like phenotypes (Shao et al., 2018). After chronic treatment with SSRIs, miR-16 levels increase in the serotonergic dorsal raphe nucleus, while its expression decreases in the noradrenergic locus coeruleus and the hippocampus (Baudry et al., 2010; Launay et al., 2011; Yang et al., 2017).

miRNAs modulate stress responses by targeting GR–related signaling pathways, thereby contributing to a positive feedback loop that reinforces HPA axis dysregulation. Studies have shown that the upregulation of miR-124 in the hippocampus of mice exposed to CUMS is closely associated with the suppression of GR mRNA expression. miR-124 directly targets GR mRNA, inhibiting its translation and thereby modulating the negative feedback regulation of GC signaling. During the early phase of stress exposure (weeks 5–6), elevated miR-124 expression is considered a compensatory response to increased GC levels (Huang et al., 2019). Another study further demonstrated that in key emotion-regulating brain regions, including the basolateral amygdala, PFC and hippocampus, both miR-124 and its target gene FKBP5 were significantly upregulated and closely associated with depression-like behavior (Xu et al., 2017). Increased FKBP5 expression may disrupt GR stability and activity, thereby exacerbating HPA axis dysregulation and contributing to the development of depressive symptoms.

The dynamic homeostatic regulation of BDNF by miRNAs should not be overlooked. miRNAs can interfere with neurotrophic factor expression and downstream signaling pathways, altering neuronal plasticity and synaptic remodeling ability. Emerging evidence identifies miR-1, miR-10b, miR-155, and miR-191 as novel regulatory factors of BDNF, expanding the epigenetic network governing neurotrophin dynamics in MDD pathophysiology (Varendi et al., 2014). Furthermore, in a CUMS-induced murine MDD model, miR-155 levels were significantly elevated, whereas BDNF expression was markedly reduced. miR-155 directly binds to the 3′UTR of BDNF mRNA, inhibiting its translation and thereby diminishing BDNF protein levels (Huan et al., 2021). Additionally, Fiori et al. demonstrated significant upregulation of miR-204-5p, miR-320b, miR-323a-3p, and miR-331-3p in the anterior cingulate cortex and habenula of individuals with MDD. Notably, miR-323a-3p was found to directly target the 3′UTR of Erb-B2 receptor tyrosine kinase 4 (ERBB4) mRNA, suppressing its expression. ERBB4, a critical receptor tyrosine kinase in neuregulin signaling pathways, functionally intersects with BDNF-mediated synaptic plasticity. ERBB4 downregulation disrupts neurotrophin signaling cascades, contributing to depressive pathogenesis through impaired glutamatergic transmission and dendritic atrophy (Fiori et al., 2021).

MiRNAs are thought to influence the progression of MDD by targeting inflammation. For instance, miR-29a-5p has been shown to alleviate depression-like behaviors by promoting anti-inflammatory microglial M2 polarization in the PFC (Yang et al., 2024a). MiR-532-5p alleviates depression-like behaviors in CUMS-exposed mice by suppressing the expression of IL-6, interleukin-1 beta (IL-1β), TNF-α, and monocyte chemoattractant protein-1 through inhibition of the signal transducer and activator of transcription 3 pathway (Yan et al., 2020).

#### 4.3.2 CircRNAs

The stable structure and tissue-specific expression patterns of circRNAs suggest their potential utility in neuropsychiatric disorders. Several circRNAs have been shown to regulate miRNA activity through a sponge-like mechanism, thereby indirectly modulating neurotransmitter-related pathways. For instance, circRNA derived from the DYM gene (circDYM) is downregulated in the plasma of patients with MDD and positively correlates with MDD severity. Functionally, circDYM inhibits microglial activation by sequestering miR-9, and its expression is upregulated following repetitive transcranial magnetic stimulation treatment, highlighting its potential as both a diagnostic biomarker and a predictor of therapeutic response.

In addition, circHIPK3 and circTulp4 have been implicated in neuroinflammatory and neurodevelopmental processes, suggesting their involvement as epigenetic regulators in the pathogenesis of MDD. More recently, circ-UBE2K has been identified as significantly upregulated in the peripheral blood of MDD patients and in the brain tissue of MDD model mice. Predominantly expressed in microglia, circ-UBE2K binds to the nuclear protein heterogeneous nuclear ribonucleoprotein U (HNRNPU), thereby enhancing the expression of its host gene, ubiquitin-conjugating enzyme E2 K (UBE2K). This interaction promotes aberrant microglial activation and neuroinflammation, contributing to the progression of MDD (Cai et al., 2024).

#### 4.3.3 LncRNAs

lncRNAs exhibit sequence specificity and typically regulate gene expression through mechanisms such as chromatin remodeling, RNA splicing, and miRNA competition. Several lncRNAs display marked sex-specific expression patterns in female patients with MDD, particularly within the PFC. LINC00473 is significantly downregulated in female MDD patients, and its reduced expression disrupts the CREB signaling pathway, thereby impairing neuronal plasticity and stress resilience (Issler et al., 2020). In contrast, FEmale DepressiOn lncRNA (FEDORA) is significantly upregulated in depressed females, is enriched in both neurons and oligodendrocytes, and its overexpression induces depressive-like behaviors, synaptic dysfunction, and myelin abnormalities in female mice (Issler et al., 2022). Notably, neither lncRNA produced comparable effects in males, indicating a distinct sex-specific regulatory role. Additionally, plasma levels of FEDORA are positively associated with clinical response to ketamine.

### 4.4 Chromatin remodeling and MDD

Histone modifications regulate chromatin structure and gene expression through chemical alterations, whereas chromatin remodeling, an ATP-dependent mechanism involving nucleosome repositioning, directly governs chromatin accessibility. This dynamic process not only synergizes with histone modifications but may also independently perturb critical pathways in MDD. For instance, overexpression of the ACF complex and its subunit BAZ1A in the NAc is strongly associated with depressive-like behaviors. Animal studies demonstrate that CSDS upregulates the bromodomain adjacent to zinc finger domain/SWI/SNF-related matrix-associated actin-dependent regulator of chromatin subfamily A member 5 complex in the NAc, promoting nucleosome clustering at transcription start sites to block gene transcription, particularly suppressing BDNF expression and increasing MDD susceptibility (Sun et al., 2015). In contrast, the homologous protein bromodomain adjacent to zinc finger domain 1B (BAZ1B) is thought to serve as an indicator of stress resilience, as it both enhances responses to rewarding stimuli and promotes adaptive responses to aversive stimuli. Although BAZ1B expression rapidly recovers post-stress exposure, persistent behavioral abnormalities suggest chromatin remodeling may induce long-term effects via downstream gene cascades (Bielawski et al., 2019). However, the roles of ATP-dependent nucleosome remodeling complexes in MDD remain poorly understood, necessitating further investigation to elucidate their mechanistic contributions.

## 5 Epigenetic antidepressant molecular mechanisms of NPs

### 5.1 Flavonoids

Flavonoids are polyphenolic NPs characterized by a C6-C3-C6 backbone, widely distributed in fruits, vegetables, and medicinal plants. Based on structural variations, they are classified into six major subclasses: flavonols, flavanols, flavanones, flavones, isoflavones, and anthocyanins. Preclinical studies have identified specific flavonoids with antidepressant potential, demonstrating their ability to reverse depressive-like behaviors in rodent models of MDD.

Hesperidin, chemically identified as hesperetin 7-O-rutinoside, is a flavanone glycoside abundantly present in citrus fruits (Li et al., 2023b). Preclinical studies across diverse CNS disease models have demonstrated its potent pharmacological properties, including antioxidant, anti-inflammatory, and neuroprotective effects (Ikram et al., 2019; Muhammad et al., 2019; Zhu et al., 2020). In lipopolysaccharide (LPS)-induced murine models, hesperidin upregulates miRNA-132 expression in the PFC. This upregulation suppresses hyperactivation of pro-inflammatory cytokines via negative feedback mechanisms, attenuating neuroinflammation and thereby exerting antidepressant-like effects (Li et al., 2016).

Quercetin, a flavonol ubiquitously distributed in fruits, vegetables, and traditional herbal medicines, exhibits multimodal pharmacological activities such as antidepressant, anticancer, gut microbiota-modulatory, antioxidant, anti-inflammatory, neuroprotective, and HPA axis-modulating properties (Chen et al., 2022). In perimenopausal MDD models, quercetin ameliorates depressive-like behaviors by binding estrogen receptors, restoring KAT/HDAC homeostasis, and significantly enhancing H3K9ac in the hypothalamus. This epigenetic modulation alleviates MDD-related phenotypes via suppression of the endoplasmic reticulum stress inositol-requiring enzyme 1 alpha/X-box binding protein 1 (IRE1α/XBP1) pathway, reducing ferroptosis-associated lipid peroxidation and mitochondrial dysfunction (Wang et al., 2024a).

Malvidin-3′-O-glucoside (Mal-gluc), a predominant anthocyanin in *Vitis vinifera*, displays antioxidant and anti-inflammatory bioactivities. It exerts antidepressant effects by downregulating HDAC2 expression in the NAc, thereby elevating histone acetylation at the ​RAC1 promoter to enhance its transcription and protein expression. RAC1, a critical small GTPase, improves dendritic spine morphology/function, enhances synaptic plasticity, and mitigates stress-induced synaptic deficits (Wang et al., 2018).

Isoliquiritin (ISL), a major flavonoid glycoside isolated from *Glycyrrhiza uralensis* Fisch., exhibits broad-spectrum pharmacological actions encompassing antioxidant, anti-inflammatory, antifungal, antidepressant, neuroprotective, angiogenic, and cardioprotective activities (Fu et al., 2025; Luo et al., 2016; Zhang et al., 2021b). ISL upregulates miR-27a to suppress spleen tyrosine kinase (SYK) translation, reducing SYK protein levels and inhibiting NF-κB pathway activation. This cascade ultimately attenuates NLRP3 inflammasome activation, Caspase-1 cleavage, and maturation of IL-1β/GSDMD with an N-terminal fragment, thereby ameliorating pyroptosis, neuroinflammation, and depressive symptomatology (Li et al., 2021d).

Genistein, a phytoestrogen derived from *Glycine max* (L.) Merr., belongs to the isoflavone class of compounds and exhibits both estrogen-like activity and a range of neuroprotective effects due to its unique chemical structure (Shete et al., 2024). In addition to its well-documented antioxidant, anti-inflammatory, and hormonal regulatory properties, Genistein has also been identified as a natural compound with epigenetic modulatory activity. It can influence gene transcription by inhibiting the expression of DNMTs (Sundaram et al., 2018). A recent study using a CUMS model in mice demonstrated that Genistein downregulates the expression of miR-221 and miR-222 in the PFC, thereby relieving the suppression of their target gene Connexin 43 (Cx43). This leads to the restoration of glial gap junction protein expression, enhancement of synaptic plasticity, and significant alleviation of depression-like behaviors (Shen et al., 2018).

### 5.2 Alkaloids

Alkaloids represent a class of nitrogen-containing organic compounds derived from plants, characterized by diverse chemical architectures and broad-spectrum bioactivities. These phytochemicals are ubiquitously distributed in roots, stems, leaves, and fruits across botanical species, with numerous alkaloids exhibiting marked pharmacological properties including antimicrobial, anti-inflammatory, antitumor, and neuromodulatory effects.

The genus Mahonia, a traditional medicinal plant in China, synthesizes over 150 chemical constituents, predominantly alkaloids such as protoberberines (e.g., berberine, palmatine, coptisine), bisbenzylisoquinolines (e.g., tetrahydroberberine), and aporphines (He and Mu, 2015). Emerging evidence identifies Mahonia alkaloids (MA) as potent antidepressant agents. Mechanistically, MA downregulates miR-205 expression to relieve its inhibitory effect on Cx43, thereby upregulating Cx43 levels. This cascade activates the CREB/BDNF signaling pathway, enhancing neuroplasticity and neuronal functionality. Notably, gap junction dysfunction is an important pathological feature of MDD (Xia et al., 2018). Cx43 is the major gap junction protein in astrocytes. MA restores the normal function of gap junctions and regulates neurosecretory function and synaptic activity through upregulation of Cx43, which then exerts antidepressant effects (He et al., 2022).

Of particular therapeutic interest is berberine, an antimicrobial alkaloid extracted from Mahonia species (e.g., *Coptis chinensis* Franch.), clinically employed for diarrheal management. Contemporary pharmacological studies reveal its multifaceted potential in cardiovascular, neurological, and psychiatric disorders. Experimental models demonstrate berberine’s capacity to suppress miR-34b-5p and miR-470-5p activity, which subsequently upregulates BDNF expression. This miRNA-mediated transcriptional modulation stimulates hippocampal neurogenesis while ameliorating depressive behaviors in murine models (Zhan et al., 2021).

### 5.3 Terpenoids

Terpenoids, a large class of NPs composed of isoprene units (C_5_H_8_), are widely distributed across plants, fungi, and marine organisms. Based on the number of isoprene units, they are classified into subclasses including monoterpenes (C_10_), sesquiterpenes (C_15_), diterpenes (C_20_), and others. Extensive research has demonstrated that terpenoids possess significant neurotherapeutic potential, exhibiting antidepressant, anxiolytic, and cognitive-enhancing effects. Their therapeutic mechanisms involve diverse molecular pathways, including reducing oxidative stress levels, antagonizing mitochondrial apoptosis, modulating inflammatory responses, regulating neurotransmitter homeostasis, promoting BDNF signaling cascades. These multifaceted actions collectively contribute to their efficacy in treating neurological disorders through precise molecular modulation.

Nerolidol (3,7,11-trimethyl-1,6,10-dodecatrien-3-ol), a naturally occurring sesquiterpenoid alcohol belonging to the monoterpenoid family, is predominantly isolated from essential oils of *Aquilaria* Lam. and other plant species (Lei et al., 2024). This compound demonstrates diverse pharmacological activities, including anti-inflammatory, antioxidative, neuroprotective, anxiolytic, and hippocampal repair properties, and has been traditionally employed in herbal medicine to alleviate fatigue, enhance qi-blood circulation, and restore mental homeostasis. At the molecular level, Nerolidol exerts antidepressant effects by significantly reducing DNMT1 expression in the brains of CUMS-induced depressed mice. Through downregulation of DNMT1, it suppresses microglial activation and attenuates the release of proinflammatory cytokines, thereby alleviating neuroinflammation (Zhang et al., 2024b).

Geniposide, a bioactive iridoid glycoside extracted from the fruits of *Gardenia jasminoides* J. Ellis, has garnered significant attention for its diverse pharmacological properties. Accumulating evidence from *in vitro* and *in vivo* studies supports its multifaceted biological activities, including neuroprotection, hepatoprotection, anti-inflammation, analgesia, antidepressant effects, cardioprotection, antioxidation, immune modulation, antithrombotic activity, and antitumor potential (Choi et al., 2024; Liu et al., 2022; Ma et al., 2024; Qin et al., 2023; Zhu et al., 2024). A recent investigation elucidates a novel molecular mechanism underlying its antidepressant efficacy: Geniposide upregulates the expression of transcription factors CREB1 and lncRNA Six3os1, thereby enhancing synaptic protein synthesis (e.g., Htr3a and Htr2a), which ultimately modulates neuronal function and ameliorates MDD-related behaviors (Li et al., 2023a).

Genipin, a monoterpenoid compound extracted from *Gardenia jasminoides* Ellis, functions as the aglycone of Geniposide. It is generated through deglycosylation in the intestine and liver, exhibiting pleiotropic bioactivities including antidepressant, anti-inflammatory, antioxidative, and neuroprotective effects. Distinct from Geniposide’s mechanism, Genipin inhibits DNMT1 activity to reduce DNA methylation at the BDNF promoter region, thereby alleviating prenatal stress-induced depressive-like behaviors through epigenetic regulation of neurotrophin synthesis (Ye et al., 2018).

Eucalyptol, a natural monoterpene predominantly derived from *Eucalyptus robusta* Sm. (Hoch et al., 2023). This aromatic compound, widely employed in food flavoring, perfumery, and pharmaceuticals, demonstrates significant anti-inflammatory, analgesic, antimicrobial, and antioxidative properties (Yin et al., 2020; Yu et al., 2019). ​Crucially, recent studies have elucidated its antidepressant efficacy via miRNA-mediated epigenetic pathways. ​Eucalyptol suppresses the expression of miR-329 and miR-362, two miRNAs that target the mRNA of brain-specific angiogenesis inhibitor 1-associated protein 3 (Baiap3) ​–a C2-domain containing protein critical for dense core vesicle (DCV) trafficking.​ ​By restoring Baiap3 expression, Eucalyptol enhances DCV-mediated 5-HT secretion, ultimately ameliorating depression-like phenotypes (Kim et al., 2021a).

Cannabidiol (CBD), a non-psychotropic phytocannabinoid isolated from the flower and leaf tissues of *Cannabis sativa* L., has garnered significant attention for its therapeutic potential in mental health disorders due to its absence of psychoactive and hallucinogenic properties (Castillo-Arellano et al., 2023). This multifaceted compound modulates CNS function through diverse neurobiological mechanisms, including antioxidant, anti-inflammatory, and protein homeostasis-regulating activities (Dash et al., 2021). In the context of antidepressant action, CBD reverses the upregulation of miR-16 and miR-135 in the PFC of CUMS-induced models, thereby alleviating depression-like behaviors through epigenetic normalization; second, it activates 5-HT1A receptors to counteract CUMS-induced transcriptional repression of the htr1a gene, ultimately enhancing serotonergic system function (Bright and Akirav, 2023).

Shanzhiside methylester (SM), a cyclohexenyl ether glycoside extracted from *Gardenia jasminoides* J. Ellis, functions as a small-molecule glucagon-like peptide-1 receptor agonist with potent anti-inflammatory, analgesic, and antidepressive properties (Fan et al., 2016). SM exerts anti-depressant effects through multiple mechanisms, with epigenetic regulation serving as a critical pathway. SM binds to miRNA-155-5p, thereby inhibiting its targeting of Suppressor of Cytokine Signaling 1 (SOCS1) mRNA and upregulating SOCS1 protein expression. The upregulation of SOCS1 subsequently suppresses janus kinase 2/signal transducer and activator of transcription 3 (JAK2/STAT3) signaling pathway activation, reduces proinflammatory cytokine production, and ultimately alleviates inflammatory responses and depressive behaviors (Sun et al., 2022).

Morroniside, a cyclohexenyl ether glycoside isolated from *Cornus officinalis* Siebold & Zucc., exhibits pleiotropic bioactivities including antioxidative, antiapoptotic, anti-inflammatory, and neuroprotective effects (Liu et al., 2021; Shi et al., 2024; Zhang et al., 2024a). Morroniside downregulates miRNA-409-3p expression, thereby derepressing BDNF transcription through the release of its 3′UTR binding site. This derepression activates the canonical BDNF/TrkB signaling cascade, which sequentially phosphorylates downstream effectors including Akt, ERK1/2, glycogen synthase kinase-3 beta, β-catenin, and CREB. The resultant upregulation of these kinases enhances neuronal survival and synaptic plasticity through multiple pathways, ultimately alleviating depressive-like phenotypes in rodent models (Qian et al., 2024).

### 5.4 Phenolic compounds

Phenolic compounds, a structurally diverse class of plant secondary metabolites characterized by hydroxyl (-OH) substituents on aromatic rings, encompass two major subclasses: phenolic acids and polyphenols. These bioactive molecules are ubiquitously present in plant tissues across various food matrices (fruits, vegetables, cereals) and medicinal herbs. Their multifaceted biological activities, including potent antioxidant, anti-inflammatory, and neuroprotective properties, are primarily attributed to their redox-active phenolic hydroxyl groups, making them promising therapeutic targets for MDD.

Dihydrocaffeic acid (DHCA), a phenolic acid containing a catechol moiety and a propyl side chain, belongs to the phenolic acids subgroup within the polyphenol family. This bioactive compound, characterized by extremely low natural abundance, is primarily found in fermented food systems and selectively accumulated in coffee extracts as a metabolic derivative of caffeic acid (CAA) and chlorogenic acid metabolism. It has a variety of biological activities such as antioxidant, anti-inflammatory and immunomodulation (Zieniuk, 2023). At the molecular level, DHCA exerts its antidepressant effects through epigenetic regulation of proinflammatory pathways. It directly inhibits DNMT1 activity, leading to hypomethylation of CpG dinucleotides in IL-6 gene introns 1 and 3. This epigenetic modification reduces IL-6 protein expression, thereby lowering peripheral inflammatory levels and exerting antidepressive effects (Wang et al., 2018).

CAA, 3,4-dihydroxycinnamic acid, a natural hydroxycinnamic acid containing phenolic and acrylic functional groups, is ubiquitously distributed in various plant matrices including coffee beans, argan oil, barley, olive oil, and selected fruits. This bioactive compound exhibits prominent antioxidant, anti-inflammatory, antimicrobial, and neuroprotective properties (Khan et al., 2021; Li et al., 2024; Pavlíková, 2022). CAA modulates the expression of epigenetic enzymes involved in DNA methylation and hydroxymethylation (including DNMT1, DNMT3a, and TET1-3), thereby affecting the transcription of BDNF and catechol-O-methyltransferase (COMT) genes to exert antidepressant effects. Additionally, CAA downregulates COMT expression, elevates dopamine levels in the brain, and ameliorates depressive symptoms through dopaminergic neurotransmission enhancement (Hu et al., 2020).

Apple phenolic extracts (APEs), a complex mixture of polyphenolic compounds derived from *Malus pumila* Mill., exhibit pleiotropic bioactivities including antioxidant, anti-inflammatory, and antiapoptotic properties (Cambeiro-Pérez et al., 2022). These bioactive components are primarily composed of chlorogenic acid, procyanidin B2, epicatechin, phloridzin, and phloretin (Feng et al., 2021). Its antidepressant mechanism involves the miR-22-3p/Sirtuin 1 (SIRT1) axis. By downregulating the level of miR-22-3p, it upregulates the expression of the NAD^+^-dependent HDAC SIRT1, thereby enhancing cellular antioxidant capacity, inhibiting the activation of inflammatory signaling pathways, and reducing cell apoptosis, thus alleviating depressive symptoms (Ren et al., 2022).

Gastrodin (GAS), a phenolic glycoside isolated from the rhizomes of *Gastrodia elata* Bl., has emerged as a promising therapeutic candidate for inflammation-associated neurological disorders due to its remarkable efficacy and safety profile (Wang et al., 2024b). Mechanistically, GAS exerts anti-depressant effects through miRNA-mediated epigenetic regulation: It downregulates miR-107-3p expression, thereby upregulating its target gene karyopherin alpha 1, reducing the production of inflammatory factors, and alleviating inflammatory response and depressive-like behavior (Song et al., 2022).

Resveratrol, a polyphenolic found in plants such as *Vitis vinifera* L. *and Reynoutria japonica* Houtt., exhibits multiple biological activities, including antioxidant, anti-inflammatory, and neuroprotective effects (Caruso et al., 2022; Ungurianu et al., 2023). As a SIRT1 activator, it regulates histone modifications through NAD^+^-dependent deacetylation, enhancing chromatin accessibility and promoting the transcriptional expression of neurotrophic factors like BDNF, highlighting its epigenetic antidepressant potential (Lagouge et al., 2006). Notably, resveratrol also upregulates the RNA-binding protein ELAV-like RNA binding protein 4 (ELAVL4), which stabilizes BDNF mRNA and improves neuroplasticity. In the CUMS model, resveratrol significantly restores neuronal morphology and dendritic spine density in the hippocampal cornu ammonis 1 (CA1) region (Ge et al., 2025). Further studies in a prenatal X-ray exposure-induced MDD model showed that resveratrol, by activating SIRT1, reverses the transcriptional repression of tryptophan hydroxylase (TPH) 2, restores 5-HT synthesis, downregulates aging-associated epigenetic markers (*p16/p21*), and upregulates BDNF, thereby alleviating depression-like symptoms (Zhang et al., 2025).

Curcumin, a natural polyphenolic compound derived from *Curcuma longa* L., belongs to the diarylheptanoid class and is widely studied for its significant antioxidant and anti-inflammatory properties (Bhat et al., 2019; Pulido-Moran et al., 2016). A study using a LPS-induced MDD rat model found that preventive administration of curcumin (40 mg/kg, intraperitoneal injection, for 7 consecutive days) effectively reversed the abnormal upregulation of miR-146a-5p in the hippocampal CA1 region. This microRNA is primarily released by activated microglia through exosomes and plays a role in inflammatory signaling regulation. Overexpression of miR-146a-5p inhibits the phosphorylation of the extracellular signal-regulated kinase (ERK) pathway, leading to the downregulation of synaptic proteins, which results in reduced dendritic spine density and synaptic dysfunction, manifesting as prominent depression-like behaviors. Curcumin intervention restores synaptic density and function by inhibiting the miR-146a-5p/ERK signaling axis, thereby producing rapid and significant antidepressant effects (Fan et al., 2021).

### 5.5 Saponins

Saponins, a class of amphiphilic NPs, are characterized by hydrophobic triterpenoid or steroidal aglycone cores conjugated with hydrophilic oligosaccharide chains via glycosidic bonds. Their surface-active properties enable foam formation in aqueous solutions, earning them the name “saponins”. These compounds predominantly occur in medicinal plants of families Araliaceae, Fabaceae, Umbelliferae, and Campanulaceae, serving as core bioactive components in traditional antidepressant formulations. Beyond their well-established anti-inflammatory, antioxidant, and immunoregulatory activities, recent studies reveal novel antidepressant mechanisms involving epigenetic regulation and neurotrophic factor signaling pathways.

Saikosaponin C (SSc), a triterpenoid saponin isolated from *Bupleurum chinense* Franch., exhibits multifaceted pharmacological activities including anti-tumor, anti-inflammatory, antidepressant, antioxidant, immunoregulatory, and hepatoprotective effects (Li et al., 2018). SSc inhibits DNMT1 activity, leading to reduced methylation of the IL-6 gene intron region and subsequent downregulation of IL-6 expression. This process disrupts the activation of the IL-6/STAT3 signaling pathway, alleviating neuroinflammation by suppressing proinflammatory mediators, while enhancing synaptic plasticity ultimately ameliorates depression-like behaviors (Bai et al., 2023).

Ginsenoside Rb1 (Rb1), a major bioactive component of *Panax ginseng* C.A. Meyer, is widely used for the treatment of various cardiovascular diseases. As one of the most abundant ginsenosides, Rb1 demonstrates multiple pharmacological activities including anti-fatigue, anti-inflammatory, immunoregulatory, neuroprotective, and antidepressant effects (Li et al., 2023c; Ni et al., 2022). Rb1 downregulates miR-134 expression, thereby relieves miR-134-mediated suppression of BDNF, activates BDNF-TrkB signaling pathway, enhances synaptic plasticity, and ultimately ameliorates depression-like behaviors (Wang et al., 2022).

### 5.6 Other types

Schisandrin B (SCHB), a major dibenzocyclooctadiene lignan isolated from *Schisandra chinensis* (Turcz.) Baill., is characterized by prominent anti-inflammatory, antioxidant, and neuroprotective properties (Ba et al., 2015; Luo et al., 2022). SCHB exerts therapeutic effects against MDD through a multifaceted molecular mechanism: it upregulates miR-124 expression, thereby suppressing NF-κB/TLR4/Myeloid differentiation primary response 88 (MyD88) and MAPK signaling pathways, which induces microglial M1 to M2 phenotypic conversion, reduces neuroinflammation, and ultimately ameliorates depression-like behaviors (Yang et al., 2024b).

Cinnamaldehyde (CA), 3-phenylprop-2-enal, a major bioactive aldehyde constituent of *Cinnamomum cassia* (L.) D. Don, has been traditionally employed for the management of MDD (Kim et al., 2021b). As a neuroprotective, anti-inflammatory, and analgesic agent (Bae et al., 2018; Mateen et al., 2019; Zhang et al., 2016), CA alleviates CUMS-induced depressive-like behaviors in middle-aged rats (Yao et al., 2015). CA upregulates GR expression in the testes, suppresses miR-190b transcription, restores BDNF levels, thereby enhancing neural plasticity and improving depression-like behaviors. Notably, these epigenetic modifications also prevent intergenerational transmission of MDD through GR/miR-190b/BDNF axis regulation (Gao et al., 2022).

Sulforaphane (SFN), a natural isothiocyanate compound derived from cruciferous vegetables such as *Brassica oleracea*, is the precursor of glucoraphanin. As a natural HDAC inhibitor, SFN plays a critical role in epigenetic regulation and exhibits significant antioxidant, anti-inflammatory, and antidepressant potential (Myzak et al., 2004). Animal studies have shown that SFN alleviates depression-like behaviors induced by CSDS by enhancing BDNF transcription. The underlying mechanism involves upregulation of nuclear factor erythroid 2-related factor 2 (Nrf2) and downregulation of methyl-CpG binding protein 2 (MeCP2) in microglia, thereby relieving transcriptional repression of the BDNF promoter. Additionally, SFN promotes the shift of microglia from a pro-inflammatory to an anti-inflammatory phenotype, further creating a microenvironment conducive to neuroprotection and synaptic plasticity (Tang et al., 2022).

Betaine is a natural trimethylamine compound widely found in *Beta vulgaris* L. (Zawieja and Chmurzynska, 2025). As a methyl donor, it regulates DNA methylation and neurotransmitter synthesis through one-carbon metabolism. In a rat model of hereditary generalized epilepsy with comorbid depression-like behaviors, maternal intake of a methyl-enriched diet containing betaine (15 g/kg) significantly alleviated depression-like behaviors in the offspring during adulthood. Mechanistically, this intervention upregulated the expression of DNMT1, hyperpolarization-activated cyclic nucleotide-gated channel 1 (HCN1), and tyrosine hydroxylase (TH) genes in the somatosensory cortex, hippocampus, and NAc, suggesting that betaine may enhance DNA methylation, block the binding of transcriptional repressors, relieve transcriptional repression of antidepressant-related genes, and enhance dopaminergic function in the midbrain-limbic system, thereby producing persistent antidepressant effects during critical periods of brain development (Sarkisova et al., 2023).

Trichostatin A (TSA), derived from the actinomycete *Streptomyces hygroscopicus*, is a hydroxamic acid compound and a classic HDACs inhibitor widely used in epigenetics research. TSA inhibits Class I and Class II HDACs, increasing histone acetylation levels, thereby modulating chromatin accessibility and activating the transcription of various genes involved in mood regulation. Studies have shown that TSA enhances H3K9/14 acetylation levels by inhibiting HDACs, directly activating BDNF promoter 1 transcription (Tian et al., 2010). In a repeated restraint stress model in male mice, TSA increased histone H3 acetylation levels, leading to the formation of transcriptionally active chromatin, which subsequently upregulated the expression of TPH in the midbrain. As the rate-limiting enzyme in 5-HT synthesis, increased TPH expression enhances 5-HT synthesis and neurotransmission, ultimately significantly reversing stress-induced mood depression (Kimijima et al., 2022). Another study demonstrated that the HDACs inhibitor TSA increased hippocampal H3K9, H4K5, and H4K12 acetylation levels, accompanied by a decrease in HDAC1/2/4/5 expression, restoring the transcriptional activity of antidepressant-related genes such as BDNF, and significantly reversing depression-like behaviors induced by the 5-HT1A receptor antagonist in mice (Zhu et al., 2021). In an Alzheimer’s disease model using amyloid precursor protein/presenilin 1 (APP/PS1) mice, TSA inhibited HDACs activity, increased histone acetylation levels, and downregulated Cystatin F-mediated microglial inflammation, significantly alleviating anxiety and depression-like behaviors in the APP/PS1 mice (Su et al., 2024) (Table 1).

**TABLE 1:** Summary of the antidepressant mechanisms of NPs via epigenetic modifications.

Classification	Natural product	Chemical structure	Molecular formula	CAS number	Source	*In vivo*/*in vitro*	Modeling method	Main indicators	Antidepressant mechanisms	References
Flavonoids	Hesperidin	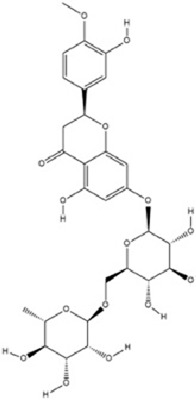	C_28_H_34_O_15_	520-26-3	Peel of citrus fruits	*In vivo*	LPS	IL-1β, IL-6, TNF-α↓; miRNA-132↑	Upregulating miRNA-132 to suppress proinflammatory cytokines via negative feedback, thereby alleviating neuroinflammation	Li et al. (2016)
Flavonoids	Quercetin	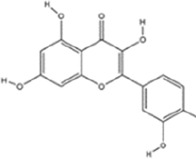	C_15_H_10_O_7_	117-39-5	Vegetables, fruits, leafy vegetables, whole grains, red wine, tea, etc.	*In vivo* and *in vitro*	*In vivo*: OVX and CUMS	N6-Acetyl-L-lysine↑; acetyl-H3K9↑	Targeting ERα/ERβ restores HAT/HDAC balance to enhance H3K9ac levels, suppress IRE1α/XBP1 activity, and reduce ferroptosis/mitochondrial dysfunction	Wang et al. (2024a)
Flavonoids	Malvidin-3′-O-glucoside	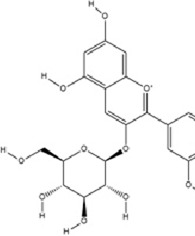	C_23_H_25_ClO_12_	7228-78-6	*Vitis vinifera*	*In vivo* and *in vitro*	*In vivo*: Repeated Social Defeat Stress, (RSDS)	Rac1, Histone acetylation↑; number of PSD-95 puncta, mEPSC frequency↓	Reducing HDAC2 in the NAc elevates histone acetylation at the Rac1 promoter to enhance its transcription and expression	Wang et al. (2018)
Flavonoids	Isoliquiritin	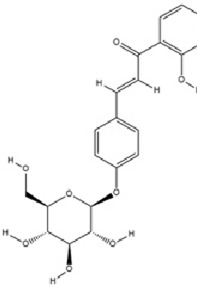	C_21_H_22_O_9_	5041-81-6	*Glycyrrhiza uralensis* Fisch.	*In vivo* and *in vitro*	*In vivo*: CSDS and LPS	miR-27a, SKY↑; p-NF-κB, IL-1β, IL-6, TNF-α↓	Upregulating miRNA-27a suppresses SYK translation, reducing NF-κB activity to inhibit NLRP3/Caspase-1/IL-1β/GSDMD-N, alleviating pyroptosis/neuroinflammation and depression	Li et al. (2021c)
Flavonoids	Genistein	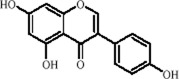	C_15_H_10_O_5_	446-72-0	*Glycine max* (L.) Merr.	*In vivo* and *in vitro*	*In vivo*: CUMS	miR-221、miR-222 expression↓, Cx43 mRNA level↑	down-regulating miR-221/222, thereby up-regulating Cx43 to restore gap-junction–mediated neuronal communication.	Shen et al. (2018)
Alkaloids	Berberine	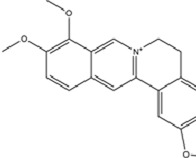	C20H18NO4+	2086-83-1	*Coptis chinensis* Franch.	*In vivo* and *in vitro*	*In vivo*: CUMS	miR-34b-5p, miR-470-5p↓; BDNF↑	Inhibiting miR-34b-5p/miR-470-5p upregulates BDNF expression to promote hippocampal neuron growth.	Zhan et al. (2021)
Alkaloids	Mahonia alkaloids	--	--	--	phylum Berberiaceae [*Mahonia bealei* (Fort.) Carr.]	*In vivo* and *in vitro*	*In vivo*: Reserpine-Induced Depression Model​	miR-205↓; Cx43↑; BDNF, p-CREB↑; 5-HT, DA, NE↑; MAO↓	inhibiting miR-205, upregulating Cx43 to restore gap junction function, and activating the CREB/BDNF signaling pathway	He et al. (2022)
Terpenoids	Nerolidol	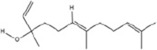	C_15_H_26_O	7212-44-4	*Aquilaria* Lam.	*In vivo* and *in vitro*	*In vivo*: CUMS	DNMT1↓; Iba-1↓; TNF-α, IL-1β, IL-6↓	Inhibiting DNMT1 to reduce microglial activation and neuroinflammation	Zhang et al. (2024b)
Terpenoids	Geniposide	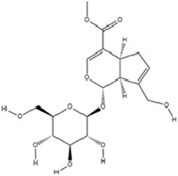	C_17_H_24_O_10_	24512-63-8	*Gardenia jasminoides* J. Ellis	*In vivo* and *in vitro*	*In vivo*: CUMS	DNMT1↓; Htr3a, Htr2a↑	Regulating the Creb1/Six3os1-synaptic protein axis to enhance synaptic plasticity	Li et al. (2023a)
Terpenoids	Genipin	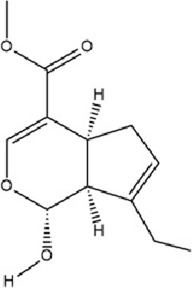	C_11_H_14_O_5_	6902-77-8	*Gardenia jasminoides* J. Ellis	*In vivo*	Prenatal Stress Model	DNMT1↓; BDNF↑	Inhibiting DNMT1 activity, upregulating BDNF expression, enhancing synaptic plasticity, and promoting neuroprotection	Ye et al. (2018)
Terpenoids	Eucalyptol	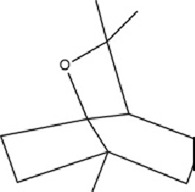	C_10_H_18_O	470-82-6	*Eucalyptus robusta* Sm.	*In vivo* and *in vitro*	*In vivo*: CUMS+ Ionizing Radiation	miR-329/362↓; Baiap3↑; 5-HT↑	Inhibiting miR-329/362, restoring Baiap3 expression, promoting DCV trafficking of 5-HT, and increasing synaptic 5-HT levels to alleviate depressive behaviors.	Kim et al. (2021a)
Terpenoids	Cannabidiol	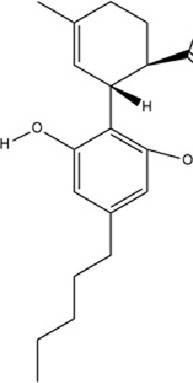	C_21_H_30_O_2_	13956-29-1	*Cannabis sativa* L.	*In vivo*​	UCMS	miR-16, miR-135↓; miR-16↑; htr1a↑	Regulating the miR-16/miR-135-5-HT1A receptor axis in the vmPFC	Bright and Akirav (2023)
Terpenoids	Shanzhiside methylester	--	--	--	*Gardenia jasminoides* J. Ellis	*In vivo* and *in vitro*	*In vivo*: CUMS	miRNA-155-5p↓; p-JAK2/p-STAT3↓, SOCS1↑, Iba1, TNF-α, IL-1β, IL-6↓	inhibiting the miRNA-155-5p/SOCS1 axis to suppress the JAK2/STAT3 signaling pathway and reduce inflammation	Sun et al. (2022)
Terpenoids	Morroniside	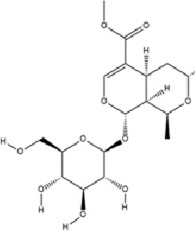	C_17_H_26_O_11_	25406-64-8	*Cornus officinalis* Siebold & Zucc.	*In vivo*​	Middle Cerebral Artery Occlusion (MCAO) combined with CUMS	miR-409-3p↓; BDNF↑; TrkB, p-AKT, p-ERK, p-GSK-3β, β-catenin, p-CREB↑	Inhibiting miR-409-3p and activating the BDNF/TrkB signaling pathway (including downstream molecules such as AKT, ERK, GSK-3β/β-catenin, and CREB)	Qian et al. (2024)
Phenolic compounds	Dihydrocaffeic acid	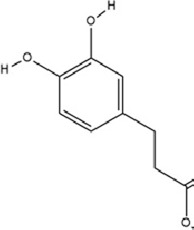	C_9_H_10_O_4_	1078-61-1	Coffea	*In vivo* and *in vitro*	*In vivo*: RSDS	DNMT1↓; IL-6↓	Inhibiting DNA methylation in the intronic regions of the IL-6 gene reduces IL-6 production and attenuates peripheral inflammation	Wang et al. (2018)
Phenolic compounds	Caffeic acid	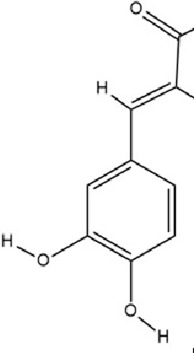	C_9_H_8_O_4_	331-39-5	Coffea	*In vivo*	CUMS	Hippocampus: 5mC↓, 5hmC↑, BDNF promoter IV mRNA levels↑; PFC: COMT mRNA levels↓	Modulating hippocampal DNA methylation (reducing 5mC and enhancing 5hmC) to restore BDNF expression, while suppressing excessive prefrontal hydroxymethylation (lowering 5hmC)	Hu et al. (2020)
Phenolic compounds	Apple Phenolic Extracts	--	--	--	*Malus pumila* Mill.	*In vivo*	Lead exposure	miR-22-3p↓; SIRT1↑	Activation of the miR-22-3p/SIRT1 signaling pathway synergistically exerts antioxidant, anti-inflammatory, and anti-apoptotic effects, thereby alleviating hippocampal damage	Ren et al. (2022)
Phenolic compounds	Gastrodin	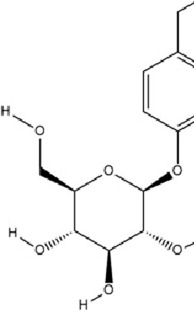	C13H18O7	62499-27-8	*Gastrodia elata* Bl.	*In vivo*	LPS	miR-107-3p↓; KPNA1 ↓; IL-1β, IL-6, TNF-α, IL-18, MCP-1↓	Downregulation of miR-107-3p and upregulation of its downstream target gene KPNA1 alleviate neuroinflammation	Song et al. (2022)
Phenolic compounds	Resveratrol	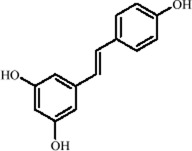	C_14_H_12_O_3_	501-36-0	*Vitis vinifera* L. and *Reynoutria japonica* Houtt.	*In vivo*	CUMS	ELAVL4↑; BDNF↑	Restoring hippocampal neuroplasticity and regulating the ELAVL4-Bdnf mRNA pathway	Ge et al. (2025)
Phenolic compounds	Resveratrol	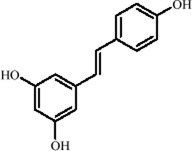	C_14_H_12_O_3_	501-36-0	*Vitis vinifera *L. and *Reynoutria japonica* Houtt.	*In vivo*	Prenatal radiation depression model	SIRT1↑; TPH2↑; 5-HT level↑	Activating SIRT1-mediated histone deacetylation to derepress the TPH2 promoter, thereby restoring 5-HT synthesis	Zhang et al. (2025)
Phenolic compounds	Curcumin	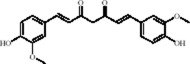	C_17_H_19_O_6_	458-37-7	*Curcuma longa* L.	*In vivo*	LPS	miR-146a-5p↓; p-ERK↑	Inhibiting miR-146a-5p overexpression, restoring ERK phosphorylation, reducing oxidative stress and neuroinflammation, inhibiting neuronal apoptosis, salvaging synaptic loss	Fan et al. (2021)
Saponins	Saikosaponin C	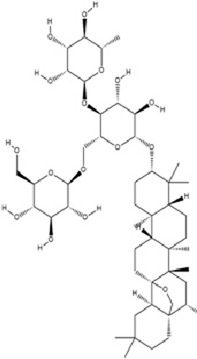	C48H78O1_7_	20736-08-7	*Bupleurum chinense* Franch.	*In vivo* and *in vitro*	*In vivo*: CSDS	DNMT1, p-STAT3/STAT3↓; IL-6, TNF-α, IL-1β↓; PSD-95↓	Inhibition of DNMT1-mediated IL-6 gene methylation reduces IL-6 expression, thereby inhibiting microglia activation and suppressing neuroinflammation; Promoting synaptic plasticity	Bai et al. (2023)
Saponins	Ginsenoside Rb1	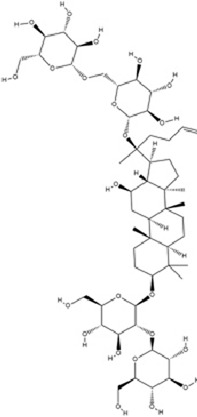	C54H92O_23_	41753-43-9	*Panax ginseng* C. A. Mey.	*In vivo*	CUMS	miR-134↓; BDNF, TrkB, p-Akt, p-ERK, p-GSK-3β (Ser9), β-catenin, p-CREB, PSD-95, GAP-43, NR2A/B, GluR1, CaMKII↑	Blocking miR-134 boosts BDNF activity, activates its TrkB signaling pathway, improves synaptic plasticity	Wang et al. (2022)
Other types	Schisandrin B	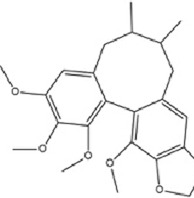	C23H28O_6_	61281-37-6	*Schisandra chinensis* (Turcz.) Baill.	*In vitro*	LPS	miR-124↑; NO, TNF-α↓; IL-10, Arg-1↑; iNOS, IBA-1↓; CD206, Arg-1↑	Upregulating miR-124 inhibits NF-κB and MAPK signaling pathways, promotes microglial polarization from the M1 to M2 phenotype, and alleviates inflammatory responses	Yang et al. (2024b)
Other types	Cinnamaldehyde	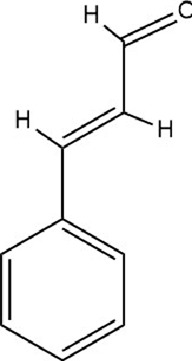	C_9_H_8_O	14371-10-9	*Cinnamomum cassia* (L.) D. Don	*In vivo* and *in vitro*	*In vivo*: CMS	GR↑; miR-190b↓; CORT↓; BDNF↑	upregulateing GR, inhibiting miR-190b, and restoring BDNF expression in the hippocampus	Gao et al. (2022)
Other types	Sulforaphane	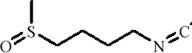	C_6_H_11_NOS_2_	4478-93-7	Cruciferous vegetables, such as *Brassica oleracea*	*In vivo* and *in vitro*	*In vivo*: CSDS	Nrf2 protein↑; MeCP2↓; BDNF↑; TNF-α, IL-1β, IL-6↓; IL-4, IL-10↑	Activating Nrf2, suppressing MeCP2, and enhancing microglial BDNF transcription, restoring dendritic spine density and mitigating inflammation to promote stress resilience	Tang et al. (2022)
Other types	Betaine	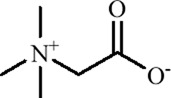	C_5_H_11_NO_2_	107-43-7	*Beta vulgaris* L.	*In vivo*	Wistar Albino Glaxo Rijswijk (WAG/Rij)	DNMT1 mRNA↑; HCN1 mRNA↑; TH mRNA↑	Through DNMT1-mediated DNA methylation reprogramming, transcriptionally up-regulating TH and HCN1, elevating mesolimbic dopaminergic tone, and suppressing cortical excitability	Sarkisova et al. (2023)
Other types	Trichostatin A	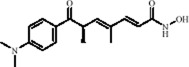	C_17_H_22_N_2_O_3_	58880-19-6	*Streptomyces platensis*	*In vivo*	Repeated restraint stress	HDAC↓; TPH↑; 5-HT↑	Inhibiting HDAC, increasing histone acetylation, enhancing the transcription of TPH gene, boosting 5-HT synthesis and release	Kimijima et al. (2022)
Other types	Trichostatin A	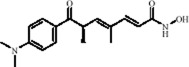	C_17_H_22_N_2_O_3_	58880-19-6	*Streptomyces platensis*	*In vivo*	Male C57BL/6 mice	HDAC1/4/5↓	Inhibiting HDACs, promoting histone acetylation, and regulating the expression of depression-related genes	Zhu et al. (2021)
Other types	Trichostatin A	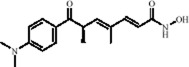	C_17_H_22_N_2_O_3_	58880-19-6	*Streptomyces platensis*	*In vivo* and *in vitro*	*In vivo*: APP/PS1; *in vitro*: LPS-induced BV2 microglial cells	HDAC↓; CST7↓	Inhibiting histone deacetylase activity, thereby increasing histone acetylation and down-regulating CST7 expression to suppress microglial inflammation	Su et al. (2024)
Other types	Sodium butyrate	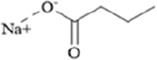	C_4_H_7_NaO_2_	156-54-7	*Faecalibacterium prausnitzii*, *Roseburia spp*., *Butyrivibrio fibrisolvens*	*In vivo*	Flinders Sensitive Line rats	TET1↑; BDNF P4 promoter 5hmC↑; BDNF P4 promoter 5 mC↓ BDNF↑	Upregulating TET1 demethylates the BDNF promoter (5mC→5hmC), relieves epigenetic repression, boosts BDNF, and enhances neuroplasticity.	Wei et al. (2015)

## 6 Toxicology and side effects

There is no bioactive compound on Earth that can simultaneously exhibit therapeutic activity and completely avoid non-specific off-target effects on normal physiological tissues (Guo et al., 2023). While NPs exhibit multi-component synergy, polytarget modulation, and epigenetic regulatory networks that offer unique advantages for MDD intervention, their complex composition simultaneously introduces potential toxicological liabilities.

The paradox of therapeutic selectivity versus systemic toxicity is exemplified by CBD, where its epigenetic modulatory effects in MDD are juxtaposed with significant safety concerns. In a clinical trial evaluating CBD’s antiepileptic properties, 12 out of 18 patients (66.7%) experienced at least one adverse event related to CBD during a 12-month treatment period with escalating doses ranging from 5 to 50 mg/kg/day. The most frequent adverse events were somnolence (44.4%, n = 8), ataxia (27.8%, n = 5), and diarrhea (22.2%, n = 4) (Hess et al., 2016). Preclinical studies further revealed dose-dependent toxicities. At 30 mg/kg, CBD caused testosterone depletionand spermatogonial cell loss in male mice, suggesting reproductive system vulnerability (Carvalho et al., 2018). Notably, 50 mg/kg CBD induced severe cardiovascular complications including hypotension and ventricular arrhythmias leading to sudden cardiac arrest in rodent models (Garberg et al., 2017).

CA demonstrates overall good tolerability with low acute toxicity, as evidenced by a wide oral LD_50_ range of 0.6–3.4 g/kg body weight in mice. Even at 20-fold higher than the effective dose (20 mg/kg), no significant behavioral or serum biochemical abnormalities were observed (Anand et al., 2010). However, chronic exposure to high doses induces dose-dependent adverse effects: marked weight loss in female rats and mice, along with histopathological alterations including esophageal squamous hyperplasia and nasal olfactory epithelial degeneration (Hooth et al., 2004). At suprapharmacological levels, CA also displays hepatotoxic potential characterized by glutathione depletion and micronucleus formation, implying genotoxic risk (Mereto et al., 1994). In the respiratory system, as an e-cigarette flavoring agent, CA causes pulmonary cytotoxicity via disruption of cellular proliferation/differentiation and induction of DNA damage in lung cells (Behar et al., 2016). Despite these findings, regulatory agencies including the Food and Drug Administration and the Council of Europe consider CA safe within the acceptable daily intake limit of 1.25 mg/kg.

In studies on the reproductive toxicity of CAA, it was found that high doses (5 and 150 mg/kg/day) affected embryo implantation, particularly when administered during the first 6 days of pregnancy, leading to reduced implantation rates. Additionally, the dose of 150 mg/kg/day also influenced fetal weight gain. The study did not observe significant maternal toxicity, teratogenic malformations, or developmental abnormalities in offspring. Meanwhile, the report proposed that the no-observed-adverse-effect level for CAA was determined to be 0.15 mg/kg/day (Liu et al., 2019b).

Geniposide, despite possessing multiple pharmacological activities, exhibits dose-dependent and duration-dependent toxicities upon high-dose or long-term administration. Studies have demonstrated that continuous administration of 300 mg/kg Geniposide induces severe hepatorenal toxicity in rats, manifesting as hepatic cellular swelling, necrosis, inflammatory infiltration, and renal tubular vacuolar degeneration. Notably, its hepatotoxicity is closely associated with the metabolic product Genipin, while thiol compounds in the liver play a crucial regulatory role in modulating its toxicity. Conversely, low-dose administration (60 mg/kg) or short-term treatment generally does not produce overt toxicity (Tian et al., 2018).

The translation challenges of NPs in drug development and clinical application primarily originate from their inherent duality of therapeutic efficacy and toxicological risks, particularly due to the coexistence of active components and endogenous toxins. Over half of natural extracts exhibit dose-dependent toxicities, manifesting not only in classical hepatorenal injuries but also emerging patterns such as cardiotoxicity and hematopoietic suppression. Furthermore, NPs exhibit diminished bioavailability due to significant first-pass metabolism and may cause delayed organ toxicity via accumulation effects. Therefore, future toxicological evaluations of NPs require continuous innovation, including advanced *in vitro*/*in vivo* assays to elucidate the mechanisms underlying hepato-renal injury. In the context of neuropsychiatric disorders, quantitative assessment of the dynamic equilibrium between antidepressant-active components and neurotoxic thresholds is critical to enable rational utilization of NPs through evidence-based frameworks.

## 7 Conclusions and prospects

MDD, as a multifaceted psychiatric disorder, presents two fundamental challenges to therapeutic intervention. First, conventional drugs such as SSRIs exhibit prolonged onset latency, marked interindividual variability in efficacy, high relapse rates post-withdrawal, and significant side effects including gastrointestinal disturbances and sexual dysfunction. Secondly, the heterogeneity of the disease itself means that its pathological mechanisms have not yet been fully elucidated, especially since the monoamine neurotransmitter hypothesis cannot explain all clinical phenomena. Recent advances in epigenetic regulation have emerged as a promising paradigm to overcome these limitations. Dysregulation of DNA methylation, histone acetylation, and ncRNA networks has been implicated in hippocampal synaptic plasticity remodeling, modulation of monoaminergic transmission, HPA axis balance, and microglia-mediated neuroinflammation, thereby illuminating the complex pathophysiology of MDD. Accordingly, this review systematically summarizes NPs-based interventions targeting epigenetic machinery, including flavonoids, alkaloids, and terpenoids, through mechanisms such as DNA methyltransferase inhibition, HDACs expression regulation, and miRNA-mRNA network modulation, which collectively enhance depressive-like behavior amelioration. Furthermore, it provides critical assessments of safety concerns related to hepatic and renal toxicity, emphasizing the necessity for precision-oriented approaches to balance epigenetic therapies’ benefits with systemic risk profiles.

Despite the promising antidepressant effects of NPs via epigenetic mechanisms in animal models, their clinical translation faces multiple challenges. First, safety remains a major concern in drug development. Most NPs exert their effects by inhibiting HDACs or DNMTs, which are widely expressed across tissues, and long-term use may lead to systemic side effects such as gastrointestinal disturbances or immune activation due to off-target toxicity. Second, the lack of target specificity limits clinical applicability. Many NPs act on multiple epigenetic enzymes, which may enhance efficacy but also increase the risk of widespread, non-specific gene expression changes and associated adverse effects. Developing more selective NPs or derivatives is therefore essential to improve therapeutic precision and reduce unwanted effects.

Additionally, NPs often exhibit dose-dependent effects, and the effective dose range varies across experimental models. For example, low-dose curcumin promotes BDNF promoter demethylation, whereas high doses may induce global methylation dysregulation. This complexity makes it difficult to define an optimal therapeutic window. Furthermore, most NPs lack selective binding to specific epigenetic enzymes or gene loci, which may lead to off-target modulation of unrelated pathways. Structural optimization and ligand-directed targeting strategies may enhance specificity.

Finally, most current studies rely on chronic stress animal models, which do not fully replicate the complexity of human MDD. Disease-specific validation is still lacking, and clinical studies on epigenetically active NPs remain scarce. Further research using patient-derived neuronal models and early-phase clinical trials is needed to assess their safety and efficacy in humans. In summary, addressing safety concerns, optimizing dosing strategies, and improving epigenetic and disease specificity are critical prerequisites for the successful clinical translation of NPs as novel antidepressant therapies.

Pharmacokinetic limitations remain a major obstacle to the clinical translation of NPs. Compounds such as resveratrol and curcumin exhibit poor water solubility, low oral bioavailability, and limited BBB permeability, reducing their effectiveness in targeting epigenetic pathways in the brain. To overcome these issues, novel delivery systems such as nanoparticles, liposomes, and transdermal formulations have been explored. For example, quercetin-loaded chitosan-coated lipid carriers showed superior antidepressant and neuroprotective effects in an LPS-induced mouse model compared to free quercetin and fluoxetine (Zewail et al., 2025). Additionally, the lack of standardized formulations and consistent quality across NP extracts hinders clinical application. Future efforts should focus on improving formulation, optimizing delivery, and evaluating pharmacokinetic–pharmacodynamic relationships to enhance therapeutic potential.

To advance the clinical translation of NPs in epigenetic antidepressant therapy, a multidimensional strategy is required. Future research should focus on improving safety, optimizing dosing, enhancing target specificity, and validating disease relevance. From a pharmacokinetic perspective, advanced delivery systems, such as nanoparticles, targeted carriers, and transdermal technologies, are needed to improve brain bioavailability. Given the complex composition and variability of NPs, efforts should also be made to standardize extraction, ensure quality control, and develop consistent formulations. Mechanistically, multi-omics, single-cell sequencing, and spatial transcriptomics can help elucidate how NPs regulate epigenetic processes in specific brain regions and cell types. In particular, region- and cell-type-specific epigenetic interventions targeting areas such as the prefrontal cortex, nucleus accumbens, or hippocampus may enhance therapeutic precision. Well-characterized NPs with favorable safety profiles should be prioritized for early clinical trials and evaluated in combination with existing antidepressants. Overall, bridging mechanistic insights with translational validation will be key to developing safe, precise, and feasible NP-based therapies for MDD.

## Glossary

MDD: Major depressive disorder
NPs: Natural products
SSRIs: Serotonin reuptake inhibitors
DNA: Deoxyribonucleic acid
HPA: Hypothalamic–pituitary–adrenal
CpG: Cytosine-phosphate-Guanine
DNMTs: DNA methyltransferases
TET: Ten-eleven translocation
NR3C1: Nuclear receptor subfamily 3 group C member 1
KATs: Lysine acetyltransferases
H3K9ac: Histone H3 lysine 9 acetylation
H3K14ac: Histone H3 lysine 14 acetylation
HMTs: Histone methyltransferases
H3K4me3: Histone H3 lysine 4 trimethylation
H3K27me: Histone H3 lysine 27 methylation
RNA: Ribonucleic acid
ncRNAs: Non-coding RNAs
lncRNAs: long non-coding RNAs
miRNAs: microRNAs
circRNAs: circular RNAs
3′UTRs: 3′untranslated regions
ATP: Adenosine triphosphate
SWI/SNF: Switching defective/sucrose nonfermenting
ISWI: Interphase structure whirlpool
INO80: Inositol requiring protein 80
CHD: Chromodomain helicase DNA-binding
CSDS: Chronic social defeat stress
NAc: Nucleus accumbens
BDNF: Brain-derived neurotrophic factor
GR: Glucocorticoid receptor
GC: Glucocorticoid
CM: Childhood maltreatment
FKBP51: FKBP5 encodes FK506-binding protein 51
SLC6A4: Solute carrier family 6 member 4
5-HT: Serotonin
AMPK/CREB: AMP-activated protein kinase/cAMP response element-binding protein
IL-6: interleukin-6
TNF-α: Tumor necrosis factor-alpha
HDACs: Histone deacetylases
PFC: Prefrontal cortex
CNS: Central nervous system
NLRP3: NOD-like receptor family pyrin domain containing 3
PI3K/Akt/mTOR: Phosphoinositide 3-kinase/protein kinase B/mammalian target of rapamycin
SYN1: Synapsin 1
TLR4: Toll-like receptor 4
H3K9me2: histone H3 lysine 9 dimethylation
RAC1: Ras-related C3 botulinum toxin substrate 1
GTPase: Guanosine triphosphatase
PCGF1: Polycomb group ring finger 1
MMP10: Matrix metallopeptidase 10
NF-κB/MAPK: Nuclear Factor kappa-light-chain-enhancer of activated B cells/mitogen-activated protein kinase
EZH2: Enhancer of zeste homolog 2
BBB: Blood-brain barrier
CDYL: Crotonyl-CoA hydratase
CUMS: Chronic unpredictable mild stress
TFIID: Transcription factor IID
H3K4me3Q5ser: Histone H3 lysine 4 trimethylation and glutaminyl-serotonylation at glutamine5
SERT: Serotonin transporter
ERBB4: Erb-B2 receptor tyrosine kinase 4
IL-1β: Interleukin-1 beta
circDYM: Circular RNA derived from the DYM gene
HNRNPU: Heterogeneous nuclear ribonucleoprotein U
UBE2K: ubiquitin-conjugating enzyme E2 K
FEDORA: FEmale DepressiOn lncRNA
BAZ1B: Bromodomain adjacent to zinc finger domain 1B
IRE1α/XBP1: Inositol-requiring enzyme 1 alpha/X-box binding protein 1
Mal-gluc: Malvidin-3′-O-glucoside
ISL: Isoliquiritin
SYK: Spleen tyrosine kinase
MA: Mahonia alkaloids
Cx43: Connexin 43
Baiap3: Brain-specific angiogenesis inhibitor 1-associated protein 3
DCV: Dense core vesicle
CBD: Cannabidiol
SM: Shanzhiside methylester
JAK2/STAT3: Janus kinase 2/signal transducer and activator of transcription 3
SOCS1: Suppressor of Cytokine Signaling 1
DHCA: Dihydrocaffeic acid
CAA: Caffeic acid
COMT: Catechol-O-methyltransferase
APEs: Apple phenolic extracts
SIRT1: Sirtuin 1
GAS: Gastrodin
ELAVL4: ELAV-like RNA binding protein 4
SSc: Saikosaponin C
Rb1: Ginsenoside Rb1
SCHB: Schisandrin B
MyD88: Myeloid differentiation primary response 88
CA: Cinnamaldehyde
LPS: Lipopolysaccharide
CA1: Cornu ammonis 1
TPH: Tryptophan hydroxylase
ERK: Extracellular signal-regulated kinase
SFN: Sulforaphane
Nrf2: Nuclear factor erythroid 2-related factor 2
MeCP2: methyl-CpG binding protein 2
HCN1: Hyperpolarization-activated cyclic nucleotide-gated channel 1
TH: Tyrosine Hydroxylase
TSA: Trichostatin A
APP/PS1: Amyloid Precursor Protein/Presenilin 1
